# Immunotoxicity of Xenobiotics in Fish: A Role for the Aryl Hydrocarbon Receptor (AhR)?

**DOI:** 10.3390/ijms22179460

**Published:** 2021-08-31

**Authors:** Helmut Segner, Christyn Bailey, Carolina Tafalla, Jun Bo

**Affiliations:** 1 Centre for Fish and Wildlife Health, Department of Pathobiology and Infectious Diseases, Vetsuisse Faculty, University of Bern, 3012 Bern, Switzerland; 2 Fish Immunology Group, CISA-INIA, 28130 Madrid, Spain; Christyn.bailey@outlook.com (C.B.); tafalla@inia.es (C.T.); 3 Laboratory of Marine Biology and Ecology, Third Institute of Oceanography, Xiamen 361005, China; bojun@tio.org.cn

**Keywords:** aryl hydrocarbon receptor, immunity, immunotoxicity, fish, environmental pollution, disease, halogenated aromatic hydrocarbons, polyaromatic hydrocarbons

## Abstract

The impact of anthropogenic contaminants on the immune system of fishes is an issue of growing concern. An important xenobiotic receptor that mediates effects of chemicals, such as halogenated aromatic hydrocarbons (HAHs) and polyaromatic hydrocarbons (PAHs), is the aryl hydrocarbon receptor (AhR). Fish toxicological research has focused on the role of this receptor in xenobiotic biotransformation as well as in causing developmental, cardiac, and reproductive toxicity. However, biomedical research has unraveled an important physiological role of the AhR in the immune system, what suggests that this receptor could be involved in immunotoxic effects of environmental contaminants. The aims of the present review are to critically discuss the available knowledge on (i) the expression and possible function of the AhR in the immune systems of teleost fishes; and (ii) the impact of AhR-activating xenobiotics on the immune systems of fish at the levels of immune gene expression, immune cell proliferation and immune cell function, immune pathology, and resistance to infectious disease. The existing information indicates that the AhR is expressed in the fish immune system, but currently, we have little understanding of its physiological role. Exposure to AhR-activating contaminants results in the modulation of numerous immune structural and functional parameters of fish. Despite the diversity of fish species studied and the experimental conditions investigated, the published findings rather uniformly point to immunosuppressive actions of xenobiotic AhR ligands in fish. These effects are often associated with increased disease susceptibility. The fact that fish populations from HAH- and PAH-contaminated environments suffer immune disturbances and elevated disease susceptibility highlights that the immunotoxic effects of AhR-activating xenobiotics bear environmental relevance.

## 1. Introduction

Immunity is a biological trait that critically relates to organism fitness [1,2]. The immune system is commonly understood as protection against non-self, such as infectious pathogens, but more broadly, it is concerned with the prevention of damage to the organism [3]. Immune responses are considered to be costly and therefore they may be traded-off with other fitness-relevant life history traits, such as reproduction, growth, metabolism, or detoxification [3,4,5]. The constituent immune activity is involved in maintaining the internal homeostasis of the organism, including the interaction with its associated microbiome [6,7]. The immune system is activated by the presence of biotic as well as abiotic stressor signals, for instance, by PAMPs (pathogen-associated molecular patterns) on the surface of invading pathogens, or by DAMPs (damage-associated molecular patterns) released from damaged tissues [8].

There is growing awareness that a variety of contaminants occurring in the aquatic environment can impact the immune system of fish species [9,10,11,12,13]. Environmental chemicals or complex matrices like, e.g., effluents of wastewater treatment plants can interfere with the constituent immunity of fish, they can modulate the pathogen-triggered activation of immune responses, and they can transiently or persistently alter the development of the immune system [13]. The observed chemical effects are most often immunosuppressive, and in a few cases also immunoactivating. Allergic reactions, as they often occur in the toxicant-exposed human immune system, have not yet been reported for toxicant-exposed fish. Chemically induced alterations of immune parameters are often classified to represent immunotoxicity, although, strictly speaking, without demonstrating that the changes are associated with impaired or dysregulated immune functioning, they may be better called an immunomodulation. However, for the sake of simplicity, we stay with the term “immunotoxicity” in the present review.

The mechanisms through which environmental chemicals interfere with the immune system of fish are little understood to date. One prominent mechanism could be the activation or inhibition of receptors and signaling pathways in the immune cells and the subsequent alteration and/or disruption of immune cell functioning. In fact, fish immune cells possess an ecosystem of membrane and intracellular receptors, which are cross-linked through diverse signaling pathways [14]. The physiological function of these receptors and signaling pathways is to orchestrate immune cell differentiation and functioning as well as to integrate the immune system with other physiological systems of the organism, such as the reproductive system or the growth axis [15]. However, the various immune system-based receptors could also be a molecular entry site through which environmental contaminants may interfere with fish immunity. An example for such a mechanism is provided by the so-called endocrine disruptors, i.e., contaminants that bind as agonists or antagonists to steroid hormone receptors in cells of the neuroendocrine and reproductive systems and thereby can disrupt fish reproduction and sexual development [15,16,17]. Steroid hormone receptors are also present in immune cells of fish [18] and consequently, estrogen- or androgen-active chemicals are able to impact not only the reproductive but also the immune system of fish [13,19].

In the present review, we focus on the possible role of the aryl hydrocarbon receptor (AhR) in mediating immunotoxic effects of aquatic contaminants in fish. Evolutionary, the AhR is present in protostomia and deuterostomia, but AhR ligand specificity and functions differ between these groups [20]. The AhR of vertebrates can be activated by a broad range of ligands, including environmental contaminants, and much of the research on the vertebrate AhR aimed to understand its role in chemical toxicity. In teleost fishes, research on the toxicity of AhR-activating contaminants has been devoted mainly to chemical biotransformation, developmental toxicity, and reproductive toxicity (e.g., [21,22,23,24]). Here, we focus on the immunotoxic activities of AhR-activating xenobiotics. Initially, we will shortly describe the canonical AhR pathway, and will summarize its discovery history. Next, we will discuss the role of the AhR in the immune system of mammals, since the immunological role of the AhR is best characterized in this vertebrate class. The next chapter will deal with the question whether the AhR is present in fish immune organs and cells. Finally, we will review what is known on the impact of AhR-activating chemicals on fish immunity at the molecular, cellular, and system levels.

The topic of this review is timely for several reasons: First, with the growing knowledge on the important physiological role of the AhR in the mammalian immune system, the question arises if this is an evolutionary conserved function and already expressed in fish. Second, while for mammals it is well documented that the binding of contaminants to the AhR causes immunotoxicity (e.g., [25,26,27,28]), we do not know if this mechanism is active in fish as well. Finally, a number of field studies have reported altered immune parameters and increased pathogen susceptibility of fish populations living in environments contaminated by AhR-activating contaminants (e.g., [9,29,30,31,32,33,34,35,36,37,38]), which raises concerns regarding the possible ecotoxicological consequences of contaminant-induced activation of the AhR in the fish immune system. 

## 2. Historical Foundation

The mammalian AhR was detected in the 1970s as a hepatic protein that binds chlorinated aromatic hydrocarbons. It could be shown that the binding correlated with the induction of a hepatic enzyme that catalyzes the chemical modification of aromatic compounds [39,40]. The binding protein was designated as an aryl hydrocarbon receptor, and the induced enzyme as aryl hydrocarbon hydroxylase, which was later identified as a cytochrome P450 (CYP) enzyme [41]. Characterization of the AhR progressed in the 1990s, when researchers succeeded in purifying the AhR protein (from rodents) and in cloning and sequencing the cDNA (cf. [42,43]). It then became clear that the AhR is a member of the bHLH (basic helix-loop-helix)-PAS (Per-Arnt-Sim) family of signal sensors [41,42]. The AhR contains an NH_2_-terminally located bHLH domain that is involved in DNA binding, a central PAS domain, and the transactivation domain towards the carboxy (COOH) terminus. The PAS domain is involved in ligand binding and interactions with chaperones (heat shock protein 90) and with the aryl hydrocarbon receptor nuclear translocator (ARNT). 

In the absence of an agonist, the cytosolic AhR is associated with chaperones like heat shock protein 90, which hold the receptor in a conformation that is able to bind a ligand. Upon ligand binding, the AhR undergoes conformational changes and translocates into the nucleus, where it heterodimerizes with ARNT. The receptor-ARNT complex finally binds to genomic regions containing its binding motif, the AHRE (aryl hydrocarbon response element, also designated as dioxin response element) and induces the transcription of target genes, for instance, biotransformation genes like CYP1A1 [42,43,44]. AhR signaling can be regulated at three levels: proteasomal degradation of the AhR, metabolic degradation of the ligands, or negative feedback regulation of the AhR-ARNT complex by the AhRR (aryl hydrocarbon receptor repressor) [45]. In addition to the canonical genomic pathway, the AhR can regulate gene expression also through non-genomic pathways [44] as well as through crosstalk with other signaling pathways like NF-κB, Nuclear Factor Erythroid-2 Related Factor 2 (nrf2), or estrogen receptors [45,46,47,48]. Related to the discovery, the originally known AhR ligands were xenobiotics. The typical xenobiotic AhR ligands include apolar planar organic compounds, such as halogenated aromatic hydrocarbons (HAHs), including dioxins, furans, and non-ortho-substituted polychlorinated biphenyls (PCBs), as well as a range of polyaromatic hydrocarbons (PAHs) [43,49]. Beyond these groups, certain polar organic compounds can also act as AhR ligands (e.g., [50]). Dioxins and related compounds enter the environment, for instance, as combustion products, as by-products in herbicides or wood preservatives, or from the bleaching process of Kraft pulp mills. PCBs have been used as lubricants and heat conductors in electrical transformers, and in a variety of other applications. Although their use has been banned, they are still present at toxic concentrations in the environment (e.g., [51]) since PCBs as well as dioxins and furans are persistent organic compounds. Environmental PAHs can originate, for instance, from oil spills, and from incomplete burning of wood, coal, or petroleum products. 

The various xenobiotic agonists display different binding affinities to the AhR, with the prototypic AhR ligand, 2,3,7,8-tetrachlorodibenzo-p-dioxin (TCDD) showing the highest binding affinity. For HAHs including TCDD, there exists a direct relationship between their binding affinity to the AhR and their toxicity in mammals [52,53]. In contrast to the knowledge on the xenobiotic agonists of the AhR, information on the nature of the physiological ligands of the AhR has developed more recently [43,45,54,55]. Among the physiological ligands, tryptophan metabolites have been identified as well as a wide array of dietary components, such as flavonoids, but also microbial molecules, including molecules produced by the symbiotic intestinal microbiota as well as molecules produced by pathogenic bacteria [56,57,58,59]. 

Much of the research on the vertebrate AhR has focused on its role as a xenobiotic sensor and its function in regulating the transcription of genes coding for xenobiotic-metabolizing enzymes, particularly the so-called AhR battery genes. In fact, due to the extensive work on the AhR, it can now serve as a working model of the functional domains and signaling steps in regulating the expression of xenobiotic-metabolizing enzymes [43]. However, the view of the AhR as a primarily xenobiotic sensor is rapidly changing. As regarded today, the AhR has multiple functions beyond toxicology, including cellular differentiation and function, coordination of the cell stress response, interaction with the intestinal microbiome, endocrine homeostasis, or longevity [27,42,43,47,57,60]. Notably, the AhR effects can vary with tissue context or ligand specificity [53]. The AhR may function as a convergence point, integrating endogenous signals as well as external signals, be they toxic chemicals, nutritional factors, or molecules of the microbiome [42,55,61,62].

## 3. The AhR in the Mammalian Immunity and Immunotoxicity

The AhR is a critical transcription factor for the normal development and function of the mammalian immune system [55,63]. The receptor is expressed in many cells of the hematopoietic lineage, and particularly in immune cells of barrier organs like skin, gut, and lungs [27,45,64]. Here, we will not provide a comprehensive review on the AhR role in the mammalian immune system, but the aim is to exemplify that the AhR has a modulating influence on a broad panel of functions in the mammalian immune system.

The AhR is involved in regulating the differentiation and function of cells of the adaptive immune system. The ligand-activated AhR influences the differentiation of CD4+ T cells into Th1, Th2, and Th17 [62,65,66,67,68]. In particular, it can alter the balance between Treg and Th17 cells [62,63]. The AhR effects appear to be ligand specific: for instance, while TCDD promotes Treg differentiation, FICZ (6-Formylindolo[3,2-b]carbazole, a natural dietary AhR ligand, induces Th17 differentiation. Treg cells express AhR, partly at levels higher than liver cells, while for Th1 and Th2 cells, low levels of AhR have been reported [26,27,55,69,70]. Through its influence on the differentiation of Treg cells, the AhR can promote anti-inflammatory actions [71,72]. The AhR-mediated induction of Treg cells has recently attracted attention for applying AhR ligands as therapeutic drugs: due to their AhR-dependent anti-inflammatory action, they may be of use in the treatment of immune-mediated diseases [73,74]. Activation of the AhR is also critical for the IL-17 and IL-22 production of Th17 cells and their role in the control of specific pathogens and in autoimmune diseases [45,55,65,71,75,76]. Finally, the AhR plays an important role in the differentiation, function, and fate of B cells [77,78,79,80]. In particular, B cells express AhR transcripts downstream of B cell receptor signaling (BCR) and the activated receptor can negatively modulate class switching and plasma cell differentiation via BLIMP1 (B-lymphocyte-induced maturation protein 1) and AICDA (activation-induced cytidine deaminase). The existence of these mechanisms has led to suggestions that the AhR could be a promising target for modulation of adaptive immune responses via vaccination [81].

In addition to its role in the adaptive immunity of mammals, the AhR has a role in controlling innate immunity as well [27,67]. The receptor is expressed at high levels in macrophages, dendritic cells, and natural killer (NK) cells. The AhR controls differentiation of monocytes into dendritic cells or macrophages, and in the macrophages, the receptor modulates the expression of pro-inflammatory cytokines as well as ROS production [70,82,83,84]. In vitro and in vivo rodent models have shown a role for the AhR in the innate immune response via macrophage differentiation during pathogen infections and promotion of IL-10 production [82,85,86]. Through these mechanisms, the AhR enhances bacterial clearance in infected mice and reduces lipopolysaccharide (LPS)-induced inflammation [87,88].

The immunological role of the AhR is particularly relevant in barrier organs like the skin or the gut as they are the first sites where the immune system encounters with exogenous AhR ligands [27,59]. In the gut, natural AhR ligands include diverse molecules contained in the diet as well as microbial molecules produced by the gut microbiome [62]. Functional AhR signaling has been shown to occur in mucosal epithelial cells of the intestine, in intraepithelial lymphocytes (IELs), and in innate lymphoid cells (ILCs), which are specialized T cells [45,55,62]. Activation of the AhR by environmental signals, such as dietary AhR ligands, has critical physiological functions in orchestrating immune homeostasis in the intestine, maintaining IEL and ILC populations, preventing inflammatory damage, and conserving barrier integrity [89]. The mechanisms through which the receptor mediates anti-inflammatory effects in the gut include a stimulating effect on the proliferation of gut-resident immune cells [45,55,59,62,90], and modulation of the gut microbiome [63,91]. The receptor also stimulates the production of the cytokine IL-22 in NK and innate lymphoid cells, and this in turn stimulates the production of antibacterial proteins by the epithelial cells [83,92]. The importance of the AhR for the mucosal immunity of mammals is also evident from the observation that in the absence of AhR activation, e.g., by feeding mice a diet containing none of the natural AhR ligands, the intestine shows increased susceptibility to bacterial infection and inflammation [63,93].

The mammalian AhR regulates through AhRE the transcription of a large number of immune genes, including complement genes and toll-like receptor genes [69,83]. The receptor also modulates the expression of pro-inflammatory cytokines like IL-6, IL-12, IFNγ, or TNFα [63,91,93]. In addition to these direct AhR-AhRE axis-mediated effects, many immune gene effects of the AhR are mediated through crosstalk with other signaling pathways like NFκB [45,48,62].

Collectively, the available data show that AhR signaling modulates a variety of mammalian immune functions, be they altered immune gene expression, modulated immune cell proliferation and differentiation, or altered immune system communication. The effects can occur directly through the AhR signaling pathway, or through interaction with other signaling pathways. The AhR is a sensor that integrates environmental, microbial, and endogenous signals in order to coordinate the immune system status under external and internal changes. Importantly, the AhR regulation of immune status and response can be cell type, tissue, and ligand specific [55]. With its integrative function, the receptor plays an important role in in maintaining immune system homeostasis and in protecting against disease [64].

Given the role of the AhR in immunity, it is not surprising that xenobiotics that act as agonists of the AhR interfere with immune functions of mammals [76]. Mostly immunosuppressive actions have been reported, typified by decreased leukocyte immune cell apoptosis, thymic atrophy, suppressed cellular immunity, disturbed T and B cell differentiation, inhibition of antibody production, and also disturbance of the intestinal microbiome and gut health [27,28,45,62,63,76,94,95,96,97,98,99]. AhR activation by xenobiotics influences not only the functioning of mature immune cells, but also the proliferation and differentiation of developing immune cells. For instance, exposure to TCDD or other AhR ligands causes an increase in Treg cells, which involves multiple mechanisms, including direct transactivation, epigenetic control of Foxp3 transcription, and modulation of dendritic cells [76]. AhR activation can also enhance autoimmune processes, e.g., through activation of inflammatory Th17 cells, and it can stimulate the secretion of the signatory inflammatory cytokine IL-22 [76,100].

In support of the laboratory findings, a series of field data point to immunosuppressive actions of xenobiotic AhR ligands. Particularly marine mammals, which bioaccumulate high levels of lipophilic PCBs and dioxins in their adipose tissues, display immunotoxic effects and suffer from increased susceptibility to infectious diseases [51,101,102,103]. For instance, recently, a relationship between PCB bioaccumulation, immune suppression, and disease mortality was shown for killer whales, *Orcinus orca.* On the basis of this relationship, the collapse of <50% of the world’s killer whale populations was predicted [101,102]. Likewise, Williams et al. [51] reported an association between blubber PCB concentrations of harbor porpoises, *Phocoena phocoena*, and increased rates of infectious disease mortality. Remarkably, this relationship existed despite the fact that the measured PCB levels were below the proposed effect thresholds. Of course, it is difficult to conclusively demonstrate in a field setting causal interference between disease incidences, immune suppression, and xenobiotic exposure; however, the pronounced immune effects of dioxins and PCBs demonstrated in controlled laboratory studies, together with the known dose–response relationships for the immunotoxic activities of these compounds, suggest a causative role of chlorinated hydrocarbons and/or PAHs in the elevated diseases incidences among marine mammals plausible.

For many of the immunotoxic effects of HAHs and PAHs in mammals, it has been shown that they are mediated either directly through the AhR pathway or through crosstalk with other signaling pathways [44,48,63,76,104,105,106,107]. The mechanistic involvement of the AhR in immunotoxic effects is also evident from studies with AhR knockout animals [98,108,109]. For AhR ligands, such as PAHs, which can be rapidly metabolized through AhR-regulated enzymes like CYP1A, the production of metabolites may also contribute to the immunotoxicity [110,111]. Importantly, more relevant than the individual, isolated immune effect could be the cascading of these single effects to the immune system level, which eventually results in the dysregulation of immune capabilities and impaired health [83,112].

This superficial overview, as summarized in Table 1, may be sufficient to highlight the critical involvement of the AhR in mammalian immunity and immunotoxicity, as well as the role of this receptor in integrating environmental and endogenous signals and translating these signals into differentiated immune outcomes [45,55,62,65]. As such, the AhR is an important factor in determining the organism’s capability to deal with variable internal and environmental conditions, including toxic contaminants.

## 4. The AhR in Fish

The AhR is present in all vertebrate classes including fish. However, as shown by the seminal work of Mark Hahn, gene and genome duplications have led to a remarkable diversification into various AhR isoforms in fish [20,113,114]. Orthologs of the mammalian AhR are present in sharks (*Chondrichthyes*), gar fish (*Holostei*), and sturgeons (*Chondrostei*), but they are absent in many teleost species. An exception is the zebrafish, where the isoform AhR1α appears to be an ortholog of the mammalian AhR [20,115]. The AhRs of teleosts can be grouped into two clades (AhR1, AhR2), with each clade often being present as duplicate pairs, i.e., AhR1a-AhR2a and AhR1b-AhR2b [20,116,117]. These pairs are thought to have arisen as part of the teleost-specific whole genome duplication [20,118]. While AhR1 was originally thought to be orthologous to the mammalian AhR, more recent analyses indicate that it represents a distinct lineage [20]. Provided that the mammalian and the teleostean AhR forms are no orthologs, this implicates that the toxicity of HAHs and PAHs is mediated trough different AhR forms in teleosts and mammals [20,21,116,117]. The various AhR forms of fish display tissue-specific expression patterns as well as differences in ligand specificity, which may lead to subfunction partitioning [20]. Moreover, Eide et al. [119] recently suggested that in fish species like many gadiform species, which have lost the xenosensor, pregnane X receptor, the AhR forms may compensate for this loss.

The essential characteristics of AhR signaling in mammals are conserved in fish, and the piscine AhRs show similar—although not identical—ligand binding preferences as the mammalian AhR, with high affinity binding of TCDD, non-ortho-substituted PCBs, and a series of PAHs [21,120,121]. The AhRs in fish have been studied intensively with respect to their role in regulating enzymes of xenobiotic metabolism and in mediating the toxicity of HAHs and PAHs, in particular their developmental and reproductive toxicity in fish [23,24,115,122,123,124,125]. The immune system as a potential target system of AhR-activating contaminants in fish, however, has attracted surprisingly little research attention. In the following sections, we will discuss the current state of knowledge on the possible physiological functions of the AhRs in fish immunity and its possible involvement in fish immunotoxicity.

## 5. The Presence and Xenobiotic Inducibility of the AhR in Immune Organs and Cells of Teleost Fish

The immune organs of teleosts include the spleen, thymus, anterior kidney (syn. pronephros/head kidney), posterior kidney (syn. mesonephros/trunk kidney/excretory kidney), and mucosa-associated lymphoid tissues (MALTs) [126,127,128,129,130,131,132,133,134]. The diffuse MALTs include the gut-associated lymphoid tissue (GALT); skin-associated lymphoid tissue (SALT); gill-associated lymphoid tissue (GIALT), which is also known as interbranchial lymphoid tissue (ILT); and the nasopharynx-associated lymphoid tissue (NALT) [135,136]. Additional MALT components have been recently identified, including the buccal MALT, pharyngeal MALT, and other might be present in the teleost eye, swim bladder, gall bladder, and stomach [132]. These tissues are composed of a variety of cell types, including B and T cells. The MALTs, together with immune cells and humoral factors present in the mucus, are essential for skin, gill epithelia, and gut function as the first immune barriers against invading pathogens [10,133]. Another important immune organ of fish is the liver [10,137]. The liver harbors diverse immune cells and about 10% of the genes annotated in the fish liver transcriptome belong to the Gene Ontology (GO) category “immune system process” [137,138]. Fish lack lymph nodes and bone marrow. Instead, in teleost fish, lymphocytopoiesis and monocytopoiesis occur in the kidneys and thymus, which are considered the primary lymphoid organs. In fish, the anterior kidney is the main organ producing B lymphocytes, while the thymus is the essential organ for the development of T lymphocytes from early thymocyte progenitors to functionally competent T cells [139]. Secondary lymphoid organs include the posterior kidney, which also has full renal capabilities, the spleen, and the MALTs [10,131].

The expression of AhR in whole immune organs has been studied in several fish species. In fish kept under control conditions in uncontaminated water, transcripts of both AhR1 and AhR2 were detected in the spleen of red seabream, *Pagrus major* [140], Atlantic salmon [141,142], and goldfish, *Carassius auratus* [143]. For the latter species, AhR1 and AhR2 protein were also present in the spleen [143]. In rainbow trout (*Oncorhynchus mykiss*), AhR mRNA was found in the head kidney and in the spleen [144,145,146]. The ratio of the expression levels of the AhR isoforms varies between tissues, for instance, in the rainbow trout spleen, the expression ratio between AhR2α and AhR2β was balanced, whereas in the heart, AhR2β transcripts showed a much higher abundance compared to AhR2α transcripts [144]. The functional implications of such tissue-specific differences in isoform ratios have not yet been elucidated.

Evolutionary history influences the AhR tissue distribution, as it may be illustrated by the case of killifish, *Fundulus heteroclitus* [147]. In populations that are sensitive to the toxic impact of HAHs and PAHs, AhR1 mRNA was expressed predominantly in the brain, heart, and gonads but not in the spleen, whereas in populations that have evolved resistance to HAH or PAH toxicity, AhR1 mRNA was present with unusually high abundances in a number of organs including the spleen [148]. For AhR2, no obvious difference between the transcript levels of resistant and sensitive killifish existed.

The developmental appearance of the AhR in fish tissues was studied by means of in situ hybridization for the Atlantic cod, *Gadus morhua* [149]. The eye was the first organ where expression of AhR was detectable (at eight days post-fertilization). The immune organs displayed no AhR staining until the end of the study period (three days post-hatching), but expression of the AhR-regulated gene, *cyp1a*, was present in a tissue, which was presumably the head kidney [149]. The inducibility of *cyp1a* transcription in the emerging head kidney suggests that the AhR signaling pathway is active early in immune system development.

Information regarding in which organs a gene product is expressed helps to deduce its physiological role. In the case of immune organs, however, findings at the whole organ level have limitations because of the heterogeneous composition of immune organs. They contain both non-immune and immune cells, and among the latter, a broad diversity of immune subpopulations and differentiation stages. Thus, studies on AhR expression at the organ level need to be broken down to studies on the expression and localization of the AhR isoforms at the level of the immune cells. Song et al. [146] compared the transcript levels of AhR2α and AhR2β in the whole head kidney organ of rainbow trout and in leukocytes isolated from the head kidney, and found similar expression levels in both systems. Additionally, the AhR induction response to BaP exposure was comparable in the whole organ and the isolated immune cells. The results from rainbow trout agree with the findings of Holen and Olsvik [150], who demonstrated by means of Western blotting that AhR protein is present in isolated head kidney leukocytes of Atlantic cod.

“Leukocytes” is a rather generic term; the question is which leukocyte subpopulations express the AhR. Song et al. [146] observed by means of fluorescent in situ hybridization (FISH) that AhR mRNA was expressed not in all but only in specific immune cell populations of rainbow trout. Although identifying immune cell types without specific cell markers is difficult, the morphological appearance of the FISH-positive cells resembled lymphocytes and neutrophils. An earlier study by Nakayama et al. [151] used double immunostaining for a key target gene of AhR signaling, CYP1A, and for surface marker proteins of trout immune cells. With this approach, the authors identified B cells and granulocytes to be CYP1A-immunoreactive. This finding would agree with the FISH data of Song et al. [146] on AhR cellular distribution. Phalen et al. [152] applied FACS sorting of rainbow trout immune cell populations, and found that AhR and CYP1A mRNA were expressed in B cells of blood, spleen, and head kidney as well as in thrombocytes of blood and spleen. Thrombocytes in fishes are nucleated cells with a primary role in blood clotting, but they appear to play a role also in the immune defense, for instance, they express genes involved in antigen presentation and they are active in phagocytosis [152,153,154].

After having confirmed that the AhR is present in fish immune cells, the next question is whether AhR expression is regulated and by which compounds. It has been reported that bacterial peptides can regulate AhR transcript levels in fish immune cells [150,155,156], but xenobiotics are also able to modulate AhR expression in fish immune organs and cells. Exposure to xenobiotic AhR agonists, such as TCDD or BaP, resulted in upregulation of AhR mRNA and protein expression in fish immune organs like the spleen [143]. In rainbow trout, BaP treatment resulted in a significant upregulation of splenic AhR2α (but not AhR22β) transcripts, whereas TCDD treatment did not lead to an induction of AhRr2α and AhR2β transcripts in the spleen [144]. Isolated head kidney leukocytes of Atlantic cod showed elevated levels of AhR protein when exposed to the PAH, phenanthrene [150]. Collectively, the available data suggest that xenobiotics—but probably also microbial peptides—can regulate AhR expression in teleostean immune cells.

The mere presence of AhR mRNA or protein in fish immune cells does not yet implicate functional AhR signaling in these cells. Evidence that the AhR pathway is functional comes from the fact that a key target gene of the AhR, CYP1A, can be upregulated in fish immune cells treated with AhR ligands. This has been shown in a number of in vivo studies when fishes exposed to compounds, such as TCDD or BaP, showed increased CYP1A immunoreactivity and/or CYP1A mRNA in immune cells [151,152,157,158,159,160]. Additionally, in vitro studies with isolated fish leukocytes show that exposure to AhR ligands results in CYP1A induction in these cells [146,150]. Importantly, CYP1A induction can be inhibited by antagonistic AhR ligands, such as α-napthoflavone [146], a finding that argues for functional involvement of the AhR signaling pathway.

In conclusion, the published data indicate that the AhR isoforms, in particular AhR2, are expressed and functional in fish immune organs and cells. One question, however, which needs much more attention in future research is which immune cell types express the AhR. The understanding of possible immunomodulatory and immunotoxicological roles of the AhR in the fish immune system depends critically on our ability to distinguish individual immune cell populations. The very preliminary data available suggest the AhR is present in B cells, thrombocytes, and granulocytes, but we have, for instance, no information on T cells or NK cells, which show high AhR abundance in mammals (see above). For a long time, the identification of AhR-positive immune cells in fish was hindered by the absence of appropriate cell markers and separation technologies. However, more recently, major progress has been made in techniques for identifying specific immune cell types of fish (e.g., [161,162,163,164,165]), and this opens ample opportunities to advance our knowledge on the cell type-specific distribution and function of the AhR in the fish immune system.

## 6. Impacts of Xenobiotic AhR Agonists on the Immunity of Teleost Fish

Absorbed toxicants are distributed within the fish organisms via the blood stream. Therefore, immune cells circulating in the blood or resident immune cells in the vascular endothelia (for instance, resident macrophages in liver sinusoids) are directly exposed to the toxic chemicals. The same applies for the highly vascularized and richly perfused immune organs. In fact, Valdez Domingos et al. [166] showed in a toxicokinetic study with medaka, *Oryzias latipes*, that a substantial fraction of absorbed PAHs is distributed to the immune organs, with the head kidney accumulating higher levels than the spleen. Likewise, Möller et al. [167] found in rainbow trout injected with 15 mg BaP/kg that the head kidney accumulated more BaP (1.3 ng/mg tissue) than the spleen (0.25 ng/mg tissue), and the accumulation in the head kidney was clearly higher than in the liver (0.4 ng/mg tissue).

Rehberger et al. [13] estimated that about 15% of the articles on fish immunotoxicology published over the last 20 years investigated the effects of AhR binding chemicals. From the findings of those studies it is evident that exposure of fish to xenobiotic AhR agonists or to environmental mixtures containing such compounds (e.g., heavy oil or creosote) can impact the immune status of fish [125,168]. In the following section, we will critically review the reports on immunological effects observed in fishes exposed to AhR-binding xenobiotics. We will structure the discussion along the biological hierarchy, i.e., from changes at the gene transcription level in immune cells and organs, over changes of cellular and systemic immune functions, to changes of systemic immunocompetence as evidenced from altered susceptibility of fish hosts to infectious pathogens (see also Table 2). As recommended by Rehberger et al. [13] and Ye et al. [138], we aim to consider modulating factors, such as gender-specific responses, or different environmental conditions, such as temperature, although the majority of published studies did not account for such variables. 

### 6.1. Immune Transcriptional Responses of Fish to AhR-Binding Xenobiotics

In vivo as well as in vitro studies reported that HAH or PAH exposure of fish or fish immune cells results in altered expression of pro-inflammatory cytokines like IL-1β, TNFα, IL-6, IL-8, or IFNγ [150,155,169,170,171,172,173]. Additionally, global transcriptomic studies corroborate that exposure to AhR ligands modulates immune gene expression of fish [170,174,175,176]. The toxicant-induced changes of immune gene transcription are not adverse by themselves, but they may have implications for the overall immune functioning of the organism. For instance, rainbow trout fed a diet containing 10 PAHs showed a reduced activation of innate immune genes under infection with *Aeromonas salmonicida* [177]. The downregulation was observed for genes encoding for complement factors and for bacterioloytic proteins, such as lysozyme. Likewise, studies on the impact of TCDD on global gene expression in whole body extracts of zebrafish, *Danio rerio*, and rainbow trout revealed a general downregulation of immune gene transcripts [178,179]. Similarly, liver transcriptomes of fishes exposed to xenobiotic AhR agonists displayed prominent changes in immune pathways (e.g., [180,181]). Additionally, the expression of genes related to the adaptive immune system is modulated by exposure to AhR ligands, for instance, an increase in the production of Th2-related transcripts [182] or a downregulation of IgM [171,183] has been observed. A prominent AhR-mediated effect in the mammalian immune system is the stimulation of IL-22 production by Th17 cells (see above). Recently, Li et al. [184] provided evidence that certain benzotriazole ultraviolet stabilizers activate *ahr2* and *cyp1a* expression in zebrafish, and through this mechanism the benzotriazole compounds modulate the expression of genes of the IL-17/IL-22 immune pathway. Collectively, these data point to immune genes as critical targets of the action of xenobiotic AhR agonists, although these observations do not yet prove that the effects are directly mediated through the AhR. PAHs and HAHs may activate alternative signaling pathways like NF-κB or NFAT signaling [169,185], or the effects may result from systemic interactions between different immune cell populations, as it has been shown in mammals.

### 6.2. Immune Functional Responses of Fish to AhR-Binding Xenobiotics

Exposure of fish to xenobiotic AhR agonists can alter the immune cell numbers and/or proliferation [30,137,145,186,187,188,189,190,191]. Likewise, exposure to complex environmental mixtures containing AhR ligands, such as creosote or PAH/PCB-contaminated sediments, has been found to induce alterations of immune cell numbers and ratios [29,36,192,193,194,195]. Unstimulated as well as mitogen-stimulated leukocytes responded to the contaminant exposure, both in vitro and in vivo. The majority of studies described that AhR ligands led to a reduction of leukocyte numbers. This can be explained by either suppressive effects on cell proliferation, as it has been described for B cells and innate immune cells [187,188,189,196,197], or by an increased rate of apoptosis [198,199,200]. However, some studies found either no leukocyte response [201,202,203,204], or even an increase of leukocyte proliferation under exposure to AhR ligands [182,186,196,197,205,206,207]. Such discrepancies might be explained by species differences and/or differences in the exposure conditions. For instance, Spitsbergen et al. [201] found a dose dependency of the TCDD immunotoxic effects, with injections of 0.1 or 1.0 µg/kg TCDD immune parameters causing no leukocyte alterations in rainbow trout but injection of 10 µg/kg TCCD suppressing immune cell proliferation. Interestingly, when leukocytes isolated from the spleen of TCDD-treated trout were stimulated with the mitogen Con-A, cell proliferation remained unaltered. However, when pokeweed mitogen (PWM) was used, they showed a proliferative response. These results indicate that the immune response is not invariate but can vary according to the treatment conditions. The currently available body of published information on the possible influence of contaminant properties, exposure dose, exposure duration, fish species, life stage, etc. on the leukocyte responses of fish under exposure to AhR ligands is by far not conclusive yet.

A particular feature of fishes are melanomacrophage centers, which are believed to have an immunological function [208]. They appear to be responsive to exposure to AhR ligands, although the existing results are controversial: while Payne and Fancey [209] found a decrease of melanomacrophage centers in the livers of flounder (*Pseudopleuronectes americanus*) exposed to PAH-contaminated sediments, van der Weiden et al. [210] observed an increase in TCDD-injected carp.

One group of immune cells that have not yet been considered in fish immunotoxico- logical studies are reticuloendothelial cells. These cells are an important component of the non-specific defense mechanisms in fish [10,211,212], and may have a prominent role particularly in the immune response to foreign particles. Exposure of fish to AhR ligands is known to result in a pronounced upregulation of the AhR target gene, cytochrome P4501A, in reticuloendothelial cells [213,214]. Therefore, the defense functions of these cells may also be sensitive to AhR ligands, but this remains to be experimentally confirmed.

Beyond changes in cell number and proliferation, direct anti-pathogenic functions of fish immune cells can be modulated by exposure to AhR-binding contaminants. In particular, oxidative burst activity, phagocytosis activity, and cytolytic activity of fish immune cells were found to be sensitive to the impact of AhR ligands [111,187,188,192,202,215,216,217,218,219]. Further immunological functions of fish that were impacted by AhR ligands including B cell functioning [188,220,221], bactericidal activity [205,222], leukocyte leukotriene secretion [155], and cytotoxic cell activity [222,223]. In addition, humoral immune parameters of fish, such as lysozyme activity, antibody levels, or complement activities, were modulated by exposure to AhR-binding xenobiotics or environmental matrices containing AhR ligands [152,192,217,223,224,225]. Generally, the toxicants had suppressive effects on the measured immune functions, although exceptions exist. For instance, an increase of the serum levels of acute-phase proteins occurred in rainbow trout exposed to TCDD, Aroclor 1254, or 3-methylcholanthrene [226]. The role of confounding factors in causing such variation of the immune responses is not well studied but must not be overlooked. For instance, Duffy and Zelikoff [227] showed that the fish immune response to coplanar PCBs is age-dependent, and White et al. [222] as well as Martin et al. [228] pointed to a marked influence of the nutritional status on the immune response. Additionally, the fact that the immune response can vary between tissues and that it is influenced by the activation status of the immune system [182,203,229,230] complicates the interpretation of the results. Finally, variations in the methodologies used to measure the immune responses of fish can be a factor contributing to contradictory findings, as it has been highlighted by Bols et al. [212] on the example of oxidative burst measurements.

An interesting case is provided by studies on PCB-resistant killifish (*Fundulus heteroclitus*) populations at highly contaminated rivers in the Eastern US. The PCB-resistant phenotypes possess a desensibilization of the AhR signaling pathway and genetic variation of the AhR genes [147]. When resistant killifish from the PCB-contaminated sites were challenged with a bacterial pathogen, they displayed normal antibacterial responses, in contrast to PCB-sensitive phenotypes, which showed a suppressed immune response. This suggests that the altered sensitivity of the AhR receptor in the PCB-resistant population provides some protection against the immunosuppressive effects of PCBs [231]. This hypothesis is supported by the toxicogenomic analysis of Ruggeri et al. [232], who showed that the killifish from the polluted sites experienced a selection of genes involved in the immune response against bacterial infections and thus may confer a genetic resistance to the immunosuppressive effects of xenobiotic AhR ligands.

### 6.3. Immune Pathology Responses of Fish to AhR-Binding Xenobiotics

Corresponding to the above-described reduction in the numbers of circulating leukocytes in fishes exposed to xenobiotic AhR agonists, a number of histopathological studies observed lymphoid depletion and hypocellularity of the lymphohematopoietic organs. Such effects have been reported, for instance, for TCDD-exposed yellow perch (*Perca flavescens*) [233], rainbow trout [210,234,235], and zebrafish [172], as well as for BaP- or DMBA-exposed tilapia [187,198]. The loss of immune cells was partly associated with an apparent increase of stromal and vascular tissue, vascular congestion, and increased immune cell apoptosis or necrosis [198,210,233,234,235,236]. Concomitant to the changes of the immune cell compartments in the spleen and kidneys, an induction of cytochrome P451A occurred, pointing to the activation of the AhR signaling pathway in the immune cells [158,159,210].

A further prominent target of the immunotoxic action of xenobiotic AhR agonists is the thymus. As noted by Grinwis et al. [158], “atrophy of the thymus and suppression of the thymus-dependent immunity is the most important immunotoxic effect of TCDD and it is seen in all animals species tested.” In teleost fish, thymus atrophy under exposure to organochlorines or PAHs has been reported from rainbow trout [234], yellow perch [233], or flounder [159].

There exists pronounced variation in the severity of the pathological lesions observed in the various studies, which probably relates to differences in the experimental conditions, such as the route of toxicant administration, toxicant dose, and exposure duration. Therefore, it is difficult to identify the causative factors of the variation in the pathological response. In addition to the previous factors, the strain of the experimental species can be of influence, as shown by Spitsbergen et al. [234], who compared the immunopathological effects of TCDD in four strains of rainbow trout. That said, it is actually surprising that the overall trend of the immunopathological responses appears to be rather uniform among fish species: lymphoid depletion and hypocellularity in the lymphohematopoietic tissues, together with thymus atrophy.

### 6.4. Altered Disease Susceptibility of Fish under Exposure to AhR-Binding Xenobiotics

If it comes to immunotoxic effects of contaminants in fish, the critical question is whether an alteration in any immune parameter would modulate the resistance of fish to infectious pathogens. As pointed out by Rehberger et al. [13], a toxicant-induced change in an immune parameter does not yet mean that the immunocompetence of the organism is compromised. The ultimate evidence to show this is provided by showing that the defense capacity of the fish host against infectious pathogens is compromised (pathogen challenge or host resistance test). A number of laboratory studies have demonstrated an increased pathogen susceptibility of fish exposed to AhR-activating contaminants. For instance, rainbow trout fed with a diet containing a PAH mixture suffered lower survival than control fish after challenge with *Aeromonas salmoncida* [237]. Similarly, Aroclor 1254 exposure reduced the resistance of Arctic char (*Salvelinus alpinus*) to *A. salmonicida* [238]. Rainbow trout embryos treated with a low PCB dose had a significantly lower survival compared to controls after bacterial infection, whereas embryos exposed to a high PCB dose showed no difference to the control group [239]. BaP treatment led to a higher susceptibility of Japanese medaka (*Oryzias latipes*) towards the pathogenic bacterium, *Yersinia ruckeri* [188]. Exposure of Japanese flounder, *Paralichtyhs olivaceus*, to heavy oil resulted in high mortalities of virus carrier fish [240].

Additionally, field studies provide evidence of an increased pathogen susceptibility of fish living in PAH and/or HAH-contaminated habitats. An example comes from the seminal studies of Arkoosh and colleagues on diseases of wild Pacific salmon living in polluted estuaries [33,35]. The elevated disease incidence appears to be a limiting factor for the recovery of Chinook salmon stocks [241]. Out-migrating juvenile Chinook salmon, *Oncorhynchus tshawytscha*, pass through contaminated urban estuaries in which they are exposed to PCBs and PAHs. These fishes display suppressed immune functions and, if challenged with infectious pathogens, they suffer elevated mortalities [34,220]. Since in a field situation it is difficult to trace back immunological alterations to a specific causative factor, Arkoosh and colleagues exposed fish in the laboratory to PCB and PAH mixtures extracted from field sediments and they could replicate the field findings on immunosuppression and increased pathogen susceptibility (cf. [35]). This provides strong evidence that the AhR-binding contaminants are indeed the etiological agent.

Another field example comes from the Deepwater Horizon oil spill, which led to the release of huge amounts of crude oil to the northern Gulf of Mexico. Following the oil spill, increased incidences of external lesions that are indicative of bacterial infections occurred among wild fish populations in the contaminated regions [242,243]. Bayha et al. [244] could confirm in a laboratory study that crude oil exposure of Southern flounder (*Paralichthys lethostigma*) results in immunosuppression and increased susceptibility to bacterial infections. Further evidence for immunotoxic effects of the Deepwater Horizon oil comes from studies with sheephead minnow, *Cyprinodon variegatus*, and red snapper, *Lutjanus campechanus* [245,246]. Collectively, field and laboratory data provide strong evidence that fish populations exposed to AhR-activating xenobiotics are particularly at risk to infectious diseases.

## 7. Concluding Remarks

### 7.1. Are There General Patterns in the Immune Response of Fish to Xenobiotic AhR Agonists?

The majority of studies on the immunological activities of xenobiotic AhR agonists in fish describe immunosuppressive effects: decreased transcript levels of immune genes, decreased leukocyte numbers, decreased leukocyte proliferation, decreased leukocyte functionality, decreased plasma humoral factors, and, associated with this, decreased pathogen resistance and reduced survival under pathogen challenge. The overall high degree of agreement in the findings of the individual studies is surprising, given the diversity of fish species and test chemicals investigated, life stages, exposure concentrations, exposure durations, and exposure routes (orally, water-borne or injection). Beyond this, the findings in fish appear to agree with those in mammals, where mostly immunosuppressive effects have also been reported for AhR-activating chemicals.

### 7.2. Are the Immunotoxic Effects of Xenobiotic AhR Agonists Mediated through the Receptor?

While numerous studies show that xenobiotic AhR agonists modulate the immune system of teleost fishes, the actual available evidence that these effects are caused directly through an AhR-dependent mechanism is limited. What has been unequivocally shown is that the AhR is present in certain immune cell populations of fishes, that treatment with AhR ligands results in the upregulation of a key AhR target gene, CYP1A, and that this induction can be inhibited by AhR antagonists, such as α-naphthoflavone (ANF) [30,146,160,171]. This indicates the existence of a functional AhR pathway in fish immune cells. However, this does not necessarily imply that AhR signaling is the key molecular event causing all known immune effects of AhR agonists.

Dysregulation of immune genes and functions directly through AhR signaling is only one possible mechanism by which environmental AhR ligands may modulate fish immunity. As discussed above, the AhR signaling pathway interacts with numerous other signaling pathways like, e.g., NF-κB, and it has been shown that TCDD and related compounds can regulate gene expression in fish via interaction with the NF-κB pathway [185,247]. AhR-independent immunotoxic mechanisms of xenobiotics may also include Ca^2+^-dependent signaling pathways, particularly in the case of PAHs [248]. Treatment of carp lymphocytes in vitro with 3-methylcholanthrene inhibited their mitogen-induced proliferation, and, in parallel, resulted in an elevation of intracellular Ca^2+^ levels [249]. Importantly, the 3-methylcholanthrene effect could not be inhibited by AhR antagonists, which argues against an AhR involvement. In the study of Hur et al. [171], the impact of BaP on TNFα gene expression could be blocked by ANF, whereas the IL-6 response was inhibited by EGTA, which suggests a mechanistic role of the Ca^2+^-dependent NFAT pathway rather than the AhR pathway. PAHs may also modulate immunity via their metabolites [160,248]. For instance, Faisal et al. [30] showed that a main metabolite of BaP, 7,8-dihydrodiol, is able to suppress immune cell mitogenesis. It is this metabolite that in rainbow trout constitutes 70% of the BaP metabolites produced in the spleen, while in the liver, 7,8-dihydrodiol accounted for only 32% of the metabolites [167]. Of course, the absolute concentration of 7,8-dihydrodiol was higher in the liver, but the local environment in the spleen appears to be dominated by this metabolite, so that an influence of this metabolite on immune functions could well be possible. Finally, we should not exclude the possibility that the environmental contaminants activate the hypothalamic-pituitary-interrenal stress axis of fish and that the resulting increase of cortisol levels could suppress immune functions [169].

**Figure 1:**
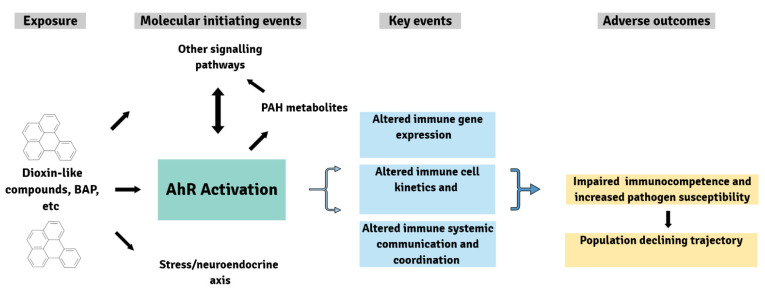
Adverse outcome pathway(s) for the immunotoxicity of AhR-activating xenobiotics. By binding to and activating the AhR as the molecular initiating event, xenobiotics potentially can dysregulate diverse molecular, cellular, and systemic key events in the immune system, and this eventually leads to compromised immunocompetence, impaired fitness (survival, reproduction), and to changes at the population level. Currently, the precise series of key events that link the molecular initiating event and the adverse outcomes is not known. Additionally, molecular initiating events alternative to AhR activation exist, which may mediate the immunotoxicity of AhR-activating xenobiotics. Still, the AOP framework can be instrumental to identify the knowledge gaps and direct future research on the immunotoxicity of AhR-activating xenobiotoics.

Concerning the current state of knowledge, the question on the mechanistic role of AhR signaling in xenobiotic immunotoxicity in fish cannot be conclusively answered. What is well documented is that chemicals activating AhR signaling modulate the immune parameters and pathogen resistance of fish, but, as often in fish toxicology, research to date stopped at the description of such correlative effects and did not analyze the underlying mechanisms and processes [250,251]. In recent years, adverse outcome pathways (AOPs) have been introduced into ecotoxicology as a framework to mechanistically connect “molecular initiating events (MIEs)” of the chemical–biological interaction through a series of “key events” to adverse effects at higher levels of biological organization [252]. There have been attempts to create such AOPs for toxic effects induced by dioxin-like chemicals in fishes, mainly for developmental, cardiac, and reproductive toxicity, but even for those well-studied toxicities of AhR-activating xenobiotics, insufficient knowledge on the key events currently prevents full AOPs being established [116]. Consequently, an AOP to link AhR activation as MIE to altered immunocompetence at the organism level of fish is not possible at the current state of knowledge. The scheme, as shown in Figure 1, therefore only presents likely but unconfirmed linkages between molecular, cellular, and systemic immune events and disease outcomes. Activation of the AhR may be one “molecular initiating event (MIE)” for the immunotoxicity of dioxin-like chemicals, whereas the immunotoxicity of PAHs may also be mediated via their metabolites, and the role of the AhR would be restricted to its influence on PAH metabolism. Apart from the question on the MIE, it is the pleiotropy of AhR-related events cascading at the system level of immunity [112] that complicates the establishment of AOPs for immunotoxic effects of xenobiotic AhR ligands.

### 7.3. Is There a Role for the Microbiome in AhR-Related Immunotoxicity in Fishes?

Over recent years, increasing evidence has accumulated that contaminants which bind to and activate the AhR can interfere with the gut microbiome of fishes [253,254,255,256]. Interestingly, the gut microbiome differs even between PAH-sensitive and PAH-tolerant populations of Atlantic killifish [257]. To date, research focused largely on the toxicant effects on the composition of the intestinal microbiome; however, from biomedical research, we know that the microbiome has an important influence on intestinal and organismic immunity [258,259,260]. If this microbiome–immunity relationship applies for fish as well, then the toxicant effects on the gut microbiome may have consequences for the immune and health status of the fish host. This question clearly warrants further research to elucidate if there is a role of the microbiome in AhR-related immunotoxicity in fishes.

### 7.4. Are the Immunotoxic Effects of AhR-Activating Contaminants Ecotoxicologically Relevant?

Many of the studies on the immunotoxicity of TCDD and related compounds in fish administrated the toxicants via injection. This raises the question of how the effects observed with this artificial administration route compare to effects in fishes naturally exposed to AhR ligands via water or diet. Beyond, it is difficult to compare concentrations on the basis of a single injection of μg or mg toxicant per kg fish to the concentrations the fishes that are chronically exposed to AhR-activating contaminants in their environment. This leads to the question of whether results from such laboratory experiments can be transferred to environmentally realistic scenarios. This is always a tricky question in ecotoxicological research. However, the evidence that immunotoxic effects are of ecological relevance is provided by the field studies: those studies show that fish populations living in habitats contaminated with HAHs and/or PAHs suffer immune suppression and increased disease susceptibility, indicating that HAH and PAH concentrations as present in the environment are indeed associated with impaired immunity.

The complication with immunotoxicity is that a chemical effect on, e.g., cytokine transcription or phagocytosis activity, is not yet adverse per se; only if it propagates into a compromised defense capacity against pathogens or reduced health it becomes ecologically relevant [261]. Although in most cases we are not yet in a position to causally connect xenobiotic-induced AhR activation, altered immunity with reduced health and pathogen resistance of fish populations, the strong association between exposure of wild fish populations to xenobiotic AhR ligands, and elevated disease outbreaks and prevalence in those populations should attract increasing attention to the immunotoxic activities of environmental contaminants. Currently, fish immunotoxicology may be in a similar situation as the endocrine disruption field was in its beginning: The trigger to attract attention to the ecotoxicological risks caused by endocrine-disrupting compounds came from field observations, not from laboratory testing. Similarly, an awareness that fish populations from habitats contaminated with AhR-activating xenobiotics suffer from impaired health and increased disease susceptibility may stimulate more research on the ecotoxicological risks of immunotoxic contaminants.

### 7.5. What Is Next?

The toxicity of AhR-activating xenobiotics in fishes has been considered primarily with respect to developmental and reproductive effects. This review highlights that these contaminants are also potent immunotoxicants to fish, which is of environmental relevance as it places fish populations at an elevated risk of pathogen infections and disease-induced mortalities. That said, it is evident that future environmental risk assessment should give more attention to the possible adverse outcomes of immunotoxic AhR ligands. Importantly, the immunotoxic effects may cumulate with the impacts of other stressors. It may be this interaction between multiple stressors, including immunotoxic chemicals, temperature change, habitat and predator stress, impaired nutrition, and hypoxia, which is responsible for the increasing emergence of infectious diseases and mass mortalities in wild fish populations [221,262,263,264,265,266,267,268].

In parallel to the future risk assessment implications, fish toxicological research needs to give more attention to understanding the mechanisms of the immunotoxic effects, and to the question to what extent the AhR activation is indeed a common molecular initiating event of the immunotoxicity of the diverse xenobiotic AhR agonists. Investigation of these questions was hindered for a long time by the absence of proper tools to study fish immunity, in particular to identify specific immune cell subpopulations, their expression of AhR family members, and their response to AhR ligands. However, with the recent advancements in fish immunology and the availability of sophisticated tools, such as transgenic fish lines or single-cell sequencing, these hindrances can be overcome. Vice versa, research on AhR-mediated immunotoxicity will advance basic knowledge of fish immunology.

## Figures and Tables

**Table 1:** Immune parameters (selection) modulated by xenobiotic AhR ligands.

Immune Cells and Tissues	Mammals	Fish
Monocytes	Regulation of monocyte differentiation (mostly) suppression of (pro)-inflammatory gene expression	not studied (mostly) suppression of (pro)-inflammatory gene expression
B cells	suppression of B cell maturation and IgM production reduced B cell proliferation	suppression of IgM production reduced B cell proliferation
T cells	regulating Treg/Th17 balance stimulation of Il-22 production by Th17 cells	Not studied stimulation of IL-22 production
Thymus	Induction of atrophy	Induction of atrophy
Mucosal immunity	Altered IEL/ILC balance Disturbance of intestinal immune homeostasis	not studied

**Table 2:** Summary of impacts of xenobiotic AhR agonists on fish immunity.

Biological Effect Level	Immune Parameters
Molecular level	altered transcription of (pro)inflammatory cytokines (mostly suppressive) altered transcription of T- and B cell immune genes (mostly suppressive)
Cellular level	altered immune cell numbers and/or proliferation (mostly suppressive) altered anti-pathogenic functions of fish immune cells (phagocytosis, oxidative burst etc.) (mostly suppressive)
Tissue level	lymphoid depletion of immune organs thymus atrophy
Organism level	Increased pathogen susceptibility and mortality
Population level	Increased disease prevalence

## Data Availability

Not applicable.

## References

[1] Lazarro B.P., Little T.J. (2009). Immunity in a variable world. Philos. Trans. R. Soc. B.

[2] Schulenburg H., Kurtz J., Moret Y., Siva-Jothy M.T.R. (2009). Introduction. Ecological immunology. Philos. Trans. R. Soc. B.

[3] Matzinger P. (2002). The danger model: A renewed sense of self. Science.

[4] Sheldon B.C., Verhulst S. (1996). Ecological immunology: Costly parasite defences and trade-offs in evolutionary ecology. Trends Ecol. Evol..

[5] Schmid-Hempl P. (2003). Variation in immune defence as a question of evolutionary ecology. Proc. R. Soc. B.

[6] Eberl G. (2010). A new vision of immunity: Homeostasis of the superorganism. Mucosal Immunol..

[7] Palmer C.V. (2018). Immunity and the coral crisis. Commun. Biol..

[8] Amadori M. (2016). The Innate Immune Response to Noninfectious Stressors: Human and Animal Models.

[9] Anderson D.P., Zeeman M.G., Rand G.M. (1995). Immunotoxicology in fish. Fundamentals of Aquatic Toxicology.

[10] Rice C.D., Schlenk D., Benson W.H. (2001). Fish immunotoxicology. Target Organ Toxicity in Marine and Freshwater Teleosts.

[11] Burnett K.G., Mommsen T.P., Moon T.W. (2005). Impacts of environmental toxicants and natural variables on the immune system of fishes. Biochemistry and Molecular Biology of Fishes.

[12] Carlson E., Zelikoff J.T., Di Giulio R.T., Hinton D.E. (2008). The Immune System of Fish. The Toxicology of Fishes.

[13] Rehberger K., Werner I., Hitzfeld B., Segner H., Baumann L. (2017). 20 years of fish immunotoxicology—What we know and where we are. Crit. Rev. Toxicol..

[14] Verburg-van Kemenade L.B.M., Cohen N., Chadzinska M. (2017). Neuroendocrine-immune interactions: Evolutionary conserved mechanisms that maintain allostasis in an ever-changing environment. Dev. Comp. Endocrinol..

[15] Villani A.C., Sarkizowa S., Hacohen N. (2018). Systems immunology: Learning the rules of the immune system. Annu. Rev. Immunol..

[16] Filby A.L., Thorpe K.L., Maack G., Tyler C.R. (2007). Gene expression profiles revealing the mechanisms of anti-androgen- and estrogen-induced feminization in fish. Aquat. Toxicol..

[17] Frye C.A., Bo E., Calamandrei G., Calza F., Dessi-Fulgheri F., Fernandez M., Fusani L., Kah O., Kajta M., LePage Y. (2012). Endocrine disruptors: A review of some sources, effects, and mechanisms of actions on behaviour and neuroendocrine systems. J. Neuroendocrinol..

[18] Casanova-Nakayama A., von Siebenthal E.W., Kropf C., Oldenberg E., Segner H. (2018). Immune-specific expression and estrogenic regulation of the four estrogen receptor isoforms in female rainbow trout (*Oncorhynchus mykiss*). Int. J. Mol. Sci..

[19] Milla S., Depiereux S., Kestemont P. (2011). The effects of estrogenic and androgenic endocrine disruptors on the immune system of fish: A review. Ecotoxicology.

[20] Hahn M.E., Karchner S.I., Merson R.R. (2017). Diversity as opportunity: Insights from 600 million years of AHR evolution. Curr. Opin. Toxicol..

[21] Hahn M.E., Hestermann E.V., Di Giulio R.T., Hinton D.E. (2008). Receptor mediated mechanisms of toxicity. The Toxicology of Fishes.

[22] Tillitt D.E., Cook P.M., Giesy J.P., Heideman W., Peterson R.E., Di Giulio R.T., Hinton D.E. (2008). Reproductive impairment of Great Lakes lake trout by dioxin-like chemicals. The Toxicology of Fishes.

[23] Segner H., Gupta R.C. (2011). Reproductive and developmental toxicity in fishes. Reproductive and Developmental Toxicology.

[24] King-Heiden T.C., Mehta V., Xiong K.M., Lanham K.A., Antkiewicz D.S., Ganser A., Heideman W., Peterson R.E. (2012). Reproductive and developmental toxicity of dioxin in fish. Mol. Cell. Endocrinol..

[25] Holsapple M.P., Snyder N.K., Wood S.C., Morris D.L. (1991). A review of 2,3,7,8-tetrachlorodibenzo-p-dioxin-induced changes in immunocompetence: 1991 update. Toxicology.

[26] Kerkvliet N.I. (2012). TCDD: An environmental immunotoxicant reveals a novel pathway of immunoregulation—A 30-year odyssey. Toxicol. Pathol..

[27] Esser C., Rannug A. (2015). The aryl hydrocarbon receptor in barrier organ physiology, immunology and toxicology. Pharmacol. Rev..

[28] Singh N.P., Nagarkatti M., Nagarkatti P. (2020). From suppressor T cells to regulatory T cells: How the journey that begun with the discovery of toxic effects of TCDD led to better understanding of the role of AhR in immunoregulation. Int. J. Mol. Sci..

[29] Weeks B.A., Warinner J.E. (1986). Functional evaluation of macrophages in fish from a polluted estuary. Vet. Immunol. Immunopathol..

[30] Faisal M., Marzouk M.S.M., Smith C.L., Huggett R.J. (1991). Mitogen-induced proliferative responses of lymphocytes from spot *(Leiostomus xanthurus*) exposed to polycyclic aromatic hydrocarbon-contaminated environments. Immunopharmacol. Immunotoxicol..

[31] Vethaak A.D., Reinallt T. (1992). Fish disease as a monitor for marine pollution: The case of the North Sea. Rev. Fish Biol. Fish..

[32] Luebke R.W., Hodson P.V., Faisal M., Ross P.S., Grasman K.A., Zelikoff J.T. (1997). Aquatic pollution-induced immunotoxicity in wildlife species. Fund. Appl. Toxicol..

[33] Arkoosh M.R., Casillas E., Huffman P., Clemons E., Evered J., Stein J.E., Varanasi U. (1998). Increased susceptibility of juvenile Chinook salmon from a contaminated estuary to *Vibrio anguillarum*. Trans. Am. Fish. Soc..

[34] Arkoosh M.R., Clemons E., Huffman P. (2001). Increased susceptibility of juvenile chinook salmon to vibriosis after exposure to chlorinated and aromatic compounds found in contaminated urban estuaries. J. Aquat. Anim. Health.

[35] Arkoosh M.R., Collier T.K. (2002). Ecological risk assessment paradigm for salmon: Analyzing immune function to evaluate risk. Hum. Ecol. Risk Assess..

[36] Khan R.A. (2003). Health of flatfish from localities in Placentia Bay, Newfoundland, contaminated with petroleum and PCBs. Arch. Environ. Contam. Toxicol..

[37] Duffy J.E., Li Y., Zelikoff J.T. (2005). PCB-induced hepatic CYP1A induction is associated with innate immune dysfunction in a feral teleost fish. Bull. Environ. Contam. Toxicol..

[38] Wojdylo J.V., Vogelbein W., Bain L.J., Rice C.D. (2013). AHR-related activities in a creosote-adapted population of adult Atlantic killifish, *Fundulus heteroclitus*, two decades post-EPA superfund status at the Atlantic Wood Site, Portsmouth, VA, USA. Aquat. Toxicol..

[39] Nebert D.W., Bausserman L.L. (1970). Fate of inducer during induction of aryl hydrocarbon hydroxylase activity in mammalian cell culture. II. Levels of intracellular polycyclic hydrocarbon during enzyme induction and decay. Mol. Pharmacol..

[40] Poland A., Glover E., Kende A.S. (1973). Stereospecific, high affinity binding of 2,3,7,8 tetra-chlorodibenzo-p-dioxin by hepatic cytosol. Evidence that the binding species is receptor for induction of aryl hydrocarbon hydroxylase. J. Biol. Chem..

[41] Okey A.B. (2007). An aryl hydrocarbon receptor odyssey to the shores of toxicology. The Deichmann lecture, International Congress of Toxicology-XI. Toxicol. Sci..

[42] Nebert D.W. (2017). Aryl hydrocarbon receptor (AHR): “pioneer member” of the basic-helix/loop/helix per-Arnt-sim (bHLH/PAS) family of “sensors” of foreign and endogenous signals. Prog. Lipid Res..

[43] Avilla M.N., Malecki K.M.C., Hahn M.E., Wilson R.H., Bradfield C.A. (2020). The Ah receptor: Adaptive metabolism, ligand diversity, and the xenokine model. Chem. Res. Toxicol..

[44] Wright E.J., Pereira de Castro K., Joshi A.D., Elferink C.J. (2017). Canonical and non-canonical aryl hydrocarbon signaling pathways. Curr. Opin. Toxicol..

[45] Stockinger B., Di Meglio P., Gialitakis M., Duarte J.H. (2014). The arylhydrocarbon receptor: Multitasking in the immune system. Annu. Rev. Immunol..

[46] Puga A., Ma C., Marlowe J.L. (2009). The aryl hydrocarbon receptor cross-talks with multiple signal transduction pathways. Biochem. Pharmacol..

[47] Guyot E., Chevallier A., Barouki R., Coumoul X. (2013). The AhR twist: Ligand-dependent AhR signalling and pharmaco-toxicological implications. Drug Discov. Today.

[48] Vogel C.F.A., Khan E.M., Leung P.S.C., Gershwin M.E., Chang W.L.W., Wu D., Haarmann-Stemmann T., Hoffmann A., Denison M.S. (2014). Cross-talk between aryl hydrocarbon receptor and the inflammatory response. J. Biol. Chem..

[49] Denison M.S., Nagy S.R. (2003). Activation of the aryl hydrocarbon receptor by structurally diverse exogenous and endogenous chemicals. Annu. Rev. Pharmacol. Toxicol..

[50] Cha J., Hong S., Lee J., Gwak J., Kim M., Kim T., Hur J., Giesy J.P., Khim J.S. (2021). Novel polar AhR-active chemicals detected in sediments of an industrial area using effect-directed analysis based on in vitro assays with full-scan high resolution mass spectrometric screening. Sci. Total Environ..

[51] Williams R., Doeschate M.T., Curnick D.J., Brownlow A., Barber J.L., Davison N.J., Deaville R., Perkins M., Jepson P.D., Jobling S. (2020). Levels of polychlorinated biphenyls are still associated with toxic effects in harbour porpoises (*Phocoena phocoena*) despite having fallen below proposed toxicity thresholds. Environ. Sci. Technol..

[52] Safe S. (1990). Polychlorinated biphenyls (PCBs), dibenzo-p-dioxins (PCDDs), dibenzofurans (PCDFs) and related compounds: Environmental and mechanistic considerations which support the development of toxic equivalency factors (TEFs). Crit. Rev. Toxicol..

[53] Birnbaum L.S. (2017). Dioxin and the AH receptor: Synergy of discovery. Curr. Opin. Toxicol..

[54] Nguyen L.P., Bradford C.A. (2007). The search for endogenous activators of the aryl hydrocarbon receptor. Chem. Res. Toxicol..

[55] Rothhammer V., Quintana F.J. (2019). The aryl hydrocarbon receptor: An environmental sensor integrating immune responses in health and disease. Nat. Rev. Immunol..

[56] Moura-Alves P., Fae K., Houthuys E., Dorhoi A., Kreuchwig A., Furkert J., Barison N., Diehl A., Munder A., Constant P. (2014). AhR sensing of bacterial pigments regulates antibacterial defence. Nature.

[57] Mulero-Navarro S., Fernandez-Salguero P. (2016). New trends in aryl hydrocarbon receptor biology. Front. Cell Dev. Biol..

[58] Murray I.A., Perdew G.H. (2017). Ligand activation of the Ah receptor contributes to gastrointestinal homeostasis. Curr. Opin. Toxicol..

[59] Lamas B., Natividad J.M., Sokol H. (2018). Aryl hydrocarbon receptor and intestinal immunity. Mucosal Immunol..

[60] Singh K.P., Casado F.L., Opanashuk L.A., Gasiewicz T.A. (2009). The aryl hydrocarbon receptor has a normal function in the regulation of hematopoietic and other stem/progenitor cell populations. Biochem. Pharmacol..

[61] Cella M., Colonna M. (2015). Aryl hydrocarbon receptor: Linking environment to immunity. Semin. Immunol..

[62] Tian J., Feng Y., Xie H.Q., Jiang J.X., Zhao B. (2015). The aryl hydrocarbon receptor: A key bridging molecule of external and internal chemical signals. Environ. Sci. Technol..

[63] Esser C., Haarmann-Stemmann T., Hochrath K., Schickowski T., Krutman J. (2018). AHR and the issue of immunotoxicity. Curr. Opin. Toxicol..

[64] Gutierrez-Vazquez C., Quintana F.J. (2018). Regulation of the immune response by the aryl hydrocarbon receptor. Immunity.

[65] Quintana F.J. (2012). The aryl hydrocarbon receptor: A molecular pathway for the environmental control of the immune response. Immunology.

[66] Quintana F.J., Sherr D.H. (2013). Aryl hydrocarbon receptor control of adaptive immunity. Pharmacol. Rev..

[67] Zhou L. (2016). AHR function in lymphocytes: Emerging concepts. Trends Immunol..

[68] Trikha P., Lee D.A. (2020). The role of AhR in transcriptional regulation of immune cell development and function. BBA Rev. Cancer.

[69] Kerkvliet N.I. (2009). AHR-mediated immunomodulation: The role of altered gene expression. Biochem. Pharmacol..

[70] Nguyen N.T., Hanieh H., Nakahama T., Kishimoto T. (2013). The roles of the arylhydrocarbon receptor in immune responses. Int. Immunol..

[71] Quintana F.J., Basso A.S., Iglesias A.H., Korn T., Farez M.F., Betelli E., Caccamo M., Oukka M., Weiner H.L. (2008). Control of Treg and TH17 cell differentiation by the aryl hydrocarbon receptor. Nature.

[72] Temchura V.V., Frericks M., Nacken W., Esser C. (2005). Role of the aryl hydrocarbon receptor in thymocyte emigration in vivo. Eur. J. Immunol..

[73] Ehrlich A.K., Kerkvliet N.I. (2017). Is chronic AhR activation by rapidly metabolized ligands safe for the treatment of immune-related diseases?. Curr. Opin. Toxicol..

[74] Kaye J., Piryatinsky V., Birnberg T., Hingaly T., Raymond E., Kashi R., Amit-Romach E., Caballero I.S., Towfin F., Ator M.A. (2016). Laquinimod arrests experimental autoimmune encephalomyelitis by activating the aryl hydrocarbon receptor. Proc. Natl. Acad. Sci. USA.

[75] Hayes M.D., Ovcinnikovs V., Smith A.G., Dearman R.J. (2014). The aryl hydrocarbon receptor: Differential contribution to T helper 17 and T cytotoxic 17 cell development. PLoS ONE.

[76] Suzuki T., Hidaka T., Kumagai Y., Yamamoto M. (2020). Environmental pollutants and the immune response. Nat. Immunol..

[77] Schneider D., Manzan M.A., Yoo B.S., Crawford R.B., Kaminski N. (2009). Involvement of Blimp-1 and AP-1 dysregulation in the 2,3,7,8-tetrachlorodibenzo-p-dioxin-mediated suppression of the IgM response by B cells. Toxicol. Sci..

[78] Sherr D.H., Monti S. (2013). The role of the aryl hydrocarbon receptor in normal and malignant B cell development. Semin. Immunopathol..

[79] Piper C.J.M., Rosser E.C., Oleinika K., Nistala K., Krausgruber T., Rendeiro A.F., Banos A., Drozdov I., Vill M., Thomson S. (2019). Aryl hydrocarbon receptor contributes to the transcriptional program of IL-10-producing regulatory B cells. Cell Rep..

[80] Blevins C.H., Zhou J., Crawford R., Kaminski N.E. (2020). TCDD-mediated suppression naive human B cell IgM secretion involves aryl hydrocarbon receptor mediated reductions in STAT3 serine 727 phosphorylation and is restored by interferon-γ. Cell Signal..

[81] Vaidyanathan B., Chaudhry A., Yewdell W.T., Angeletti D., Yen W.F., Wheatley A.K., Bradfield C.A., McDermott A.B., Yewdell J.W., Rudensky A.Y. (2017). The aryl hydrocarbon receptor controls cell-fate decisions in B cells. J. Exp. Med..

[82] Kimura A., Naka T., Nakahama T., Chinen I., Masuda K., Nohara K. (2009). Aryl hydrocarbon receptor in combination with Stat1 regulates LPS-induced inflammatory responses. J. Exp. Med..

[83] Memari B., Bouttier M., Dimitrov V., Oullette M., Behr M.A., Fritz J.H., White J.H. (2015). Engagement of the aryl hydrocarbon receptor in *Mycobacterium tuberculosis*-infected macrophages has pleiotropic effects on innate immune signaling. J. Immunol..

[84] Goudot C., Coillard A., Villani A.C., Guguen P., Cros A., Sarkizova S., Tang Huau T.L., Bohec M., Baulande S., Hacohen N. (2017). Aryl hydrocarbon receptor controls monocyte differentiation into dendritic cells versus macrophages. Immunity.

[85] Climaco-Arvizu S., Dominguez-Acosta O., Cabanas-Cortes M.A., Rodriguez-Sosa M., Gonzalez F.J., Vega L. (2016). Aryl hydrocarbon receptor influences nitric oxide and arginine production and alters M1/M2 macrophage polarization. Life Sci..

[86] Zhu J., Luo L., Tian L., Yin S., Ma X., Cheng S., Tang W., Yu J., Ma W., Zhou X. (2018). Aryl hydrocarbon receptor promotes IL-10 expression in inflammatory macrophages through Src-STAT3 signaling pathway. Front. Immunol..

[87] Shi L.Z., Faith N.G., Nakayama Y., Suresh M., Steinberg H., Czuprynski C.J. (2007). The aryl hydrocarbon receptor is required for optimal resistance to Listeria monocytogenes infection in mice. J. Immunol..

[88] Kimura A., Abe H., Tsuruta S., Chiba S., Fuji-Kuriyami Y., Sekiya T., Morita R., Yoshimura A. (2014). Aryl hydrocarbon receptor protects against bacterial infection by promoting macrophage survival and reactive oxygen species production. Int. Immunol..

[89] Metidji A., Omenetti S., Crotta S., Li Y., Nye E., Ross E., Li V., Maradana M.R., Schiering C., Stockinger B. (2018). The environmental sensor AHR protects from inflammatory damage by maintaining intestinal stem cell homeostasis and barrier integrity. Immunity.

[90] Li Y., Innocentin S., Withers D.R., Roberts N.A., Gallagher A.R., Grogrieva E.F., Wilhelm C., Veldhoen M. (2011). Exogenous stimuli maintain intraepithelial lymphocytes via aryl hydrocarbon receptor activation. Cell.

[91] Murray I.A., Nichols R.G., Zhang L., Patterson A.D., Perdew G.H. (2016). Expression of the aryl hydrocarbon receptor contributes to the establishment of intestinal microbial community structure in mice. Sci. Rep..

[92] Monteleone L., Rizzo A., Sarra M., Sica G., Sileri P., Biancone L., McDonald T.T., Pallone F., Monteleone G. (2011). Aryl hydrocarbon receptor-induced signals up-regulate IL-22 production and inhibit inflammation in the gastrointestinal tract. Gastroenterology.

[93] Goettel J.A., Gandhi R., Kenison J.E., Yeste A., Murugaiyan G., Sambanthamoorthy S., Griffith A.E., Patel B., Shouval D.S., Weiner H.L. (2016). AhR activation is protective against colitis driven by T cells in humanized mice. Cell Rep..

[94] Weng C.M., Wang C.H., Lee M.J., He R.J., Huang H.Y., Chao M.W., Chung K.F., Kuo H.P. (2018). Aryl hydrocarbon receptor activation by diesel exhaust particles mediates epithelium derived cytokines expression in severe allergic asthma. Allergy.

[95] Du F., Zhao T., Ji H.J., Luo Y.B., Wang F., Mao G.H., Feng W.W., Chen Y., Wu X.Y., Yang L.Q. (2019). Dioxin-like (DL) polychlorinated biphenyls induced immunotoxicity through apoptosis in mice splenocytes via the AhR-mediated mitochondria-dependent signaling pathways. Food Chem. Toxicol..

[96] Chen L., Zhang W., Hua J., Hu C., Lai N.L., Qian P.Y., Lam P.K.S., Lam J.W.C., Zhou B. (2018). Dysregulation of intestinal health by environmental pollutants: Involvement of the estrogen receptor and aryl hydrocarbon receptor. Environ. Sci. Technol..

[97] Cole L., Beamer Shepherd C.A., Kreitinger J., Shepherd D.M. (2018). Aryl hydrocarbon receptor activation in DCs regulates thymic function and immune tolerance induction. J. Immunol..

[98] Beamer C.A., Kreitinger J.M., Cole S.L., Shepherd D.M. (2019). Targeted deletion of the aryl hydrocarbon receptor in dendritic cells prevents thymic atrophy in response to dioxin. Arch. Toxicol..

[99] Pang C., Zhu C., Zhang Y., Ge Y., Li S., Huo S., Xu T., Stauber R.H., Zhao P. (2019). 2,3,7,8,-tetrachlorodibenzo-p-dioxin affects the differentiation of CD4 helper T cell. Toxicol. Lett..

[100] Van Voorhis M., Knopp S., Juillard W., Fechner J.H., Zhang X., Schauer J.J., Meznich J.D. (2013). Exposure to atmospheric particulate matter enhances Th17 polarization through the aryl hydrocarbon receptor. PLoS ONE.

[101] Hall A.J., McConnell B.J., Schwacke L.H., Ylitalo G.M., Williams R., Rowles T.K. (2018). Predicting the effects of polychlorinated biphenyls on cetacean populations through impacts on immunity and calf survival. Environ. Poll..

[102] Desforges J.P., Hall A., McConnell B., Rosing-Asvid A., Barber J.L., Brownlow A., De Guise S., Eulaers I., Jepson P.D., Letcher R.J. (2018). Predicting global killer whale population collapse from PCB pollution. Science.

[103] Sonne C., Siebert U., Gonnsen K., Desforges J.P., Eulaers I., Persson S., Roos A., Bäcklin B.M., Kauhala K., Olsen M.T. (2020). Health effects from contaminant exposure in Baltic Sea birds and marine mammals: A review. Environ. Int..

[104] Fueldner C., Kohlschmidt J., Riemschneider S., Schulze F., Zoldan K., Esser C., Hauschildt S., Lehmann J. (2018). Benzo(a)pyrene attenuates the pattern-recognition receptor-induced proinflammatory phenotype of murine macrophages by inducing IL-10 expression in an aryl hydrocarbon receptor-dependent way. Toxicology.

[105] O’Driscoll C.A., Mezrich J.D. (2018). The aryl hydrocarbon receptor as an immune modulator of atmospheric particulate matter-mediated autoimmunity. Front. Immunol..

[106] Tajima H., Tajiki-Nishino R., Watanabe Y., Fukuyama T. (2019). Direct activation of aryl hydrocarbon receptor by benzo(a)pyrene elicits T-helper 2-driven proinflammatory responses in a mouse model of allergic dermatitis. J. Appl. Toxicol..

[107] Li J., Phadnis-Moghe A.S., Crawford R.B., Kaminski N.E. (2017). Aryl hydrocarbon receptor activation by 2,3,7,8-tetrachlorodibenzo-p-dioxin impairs human B lymphopoiesis. Toxicology.

[108] Harrill J.A., Layko D., Nyska A. (2016). Aryl hydrocarbon receptor knockout rats are insensitive to the pathological effects of repeated oral exposure to 2,3,7,8-tetrachlorodibenzo-p-dioxin. J. Appl. Toxicol..

[109] Phadnis-Moghe A.S., Chen W., Li J., Crawford R.B., Bach A., D’Ingillo S., Kovalova N., Suarez-Martinez J.E., Kaplan B.L.F., Harrill J.A. (2016). Immunological characterization of the aryl hydrocarbon receptor (AHR) knockout mouse in the presence and absence of 2,3,7,8-tetrachlorodibenzo-p-dioxin (TCDD). Toxicology.

[110] Mann K.K., Matulka R.A., Hahn M.E., Trombino A.F., Lawrence B.P., Kerkvliet N.I., Sherr D.H. (1999). The role of polycyclic aromatic hydrocarbon metabolism in dimethyl-benz(a)anthracene-induced pre-B-lymphocyte apoptosis. Toxicol. Appl. Pharmacol..

[111] Gao J., Lauer F.T., Dunaway S., Burchiel S.W. (2005). Cytochrome P450 1B1 is required for 7,12,-dimethylbenz(a)-anthracene (DMBA) induced spleen cell immunotoxicity. Toxicol. Sci..

[112] Ambrosio L.D.F., Insfran C., Volpini X., Rodriguez E.A., Serra H.M., Quintana F.J., Cervi L., Motran C.C. (2019). Role of the aryl hydrocarbon receptor (AhR) in the regulation of immunity and immunopathology during *Trypanosoma cruzi* infection. Front. Immunol..

[113] Hahn M.E. (2002). Aryl hydrocarbon receptors: Diversity and evolution. Chem-Biol. Interact..

[114] Hahn M.E. (2006). Unexpected diversity of aryl hydrocarbon receptors in non-mammalian vertebrates: Insights from comparative genomics. J. Exp. Zool. A.

[115] Shankar P., Dasgupta S., Hahn M.E., Tanguay R.L. (2020). A review on the functional roles of the zebrafish aryl hydrocarbon receptors. Toxicol. Sci..

[116] Doering J.A., Wiseman S., Giesy J.P., Hecker M. (2018). A cross-species quantitative adverse outcome pathway fro activation of the aryl hydrocarbon receptor leading to early life stage mortality in birds and fishes. Environ. Sci. Technol..

[117] Aranguren-Abadia L., Lille-Langoy R., Maddsen A.K., Karchner S.I., Franks D.G., Yadetie F., Hahn M.E., Goksoyr A., Karlsen O.A. (2020). Molecular and functional properties of the Atlantic cod (*Gadus morhua*) aryl hydrocarbon receptors AhR1a and AhR2a. Environ. Sci. Technol..

[118] Glasauer S.M., Neuhauss S.C. (2014). Whole genome duplication in teleost fishes and its evolutionary consequences. Mol. Genet. Genom..

[119] Eide M., Rydbeck H., Torresen O.K., Lille-Langoy R., Puntervoll P., Goldstone J.V., Jakobsen J.S., Stegeman J.J., Goksoyr A., Karlsen O.A. (2018). Independent losses of a xenobiotic receptor across teleost evolution. Sci. Rep..

[120] Stegeman J.J., Hahn M.E., Malins D.C., Ostrander G.K. (1994). Biochemistry and molecular biology of monooxygenases: Current perspectives on forms, functions and regulation of cytochrome P450 in aquatic species. Aquatic Toxicology: Molecular, Biochemical and Cellular Perspectives.

[121] Hahn M.E., Merson R.R., Karchner S.I., Mommsen T.P., Moon T.W. (2005). Xenobiotic receptors in fish: Structural and functional diversity and evolutionary insights. Biochemistry and Molecular Biology of Fishes.

[122] Tanguay R.L., Andreasen E.A., Walker M.K., Peterson R.E., Schlechter A., Gasiewicz T.A. (2003). Dioxin toxicity and aryl hydrocarbon receptor signaling in fish. Dioxins and Health.

[123] Carney S.A., Prasch A.L., Heideman W., Peterson R.E. (2006). Understanding dioxin developmental toxicity using the zebrafish model. Birth Defects Res..

[124] Goldstone H., Stegeman J.J. (2006). Molecular mechanisms of 2,3,7,8-tetrachlorodibenzo-p-dioxin cardiovascular embryotoxicity. Drug Metabol. Rev..

[125] Johnson L.L., Anulacion B.F., Burrows D.G., da Silva D.A.M., Dietrich J.P., Myers M.S., Spromberg J., Ylitalo G.M. (2014). Effects of legacy persistent organic pollutants (POPs) in fish—Current and future challenges. Fish Physiol..

[126] Fänge R. (1994). Blood cells, haemopoiesis and lymphomyeloid tissues in fish. Fish Shellfish. Immunol..

[127] Press C.M., Evensen O. (1999). The morphology of the immune system in teleost fishes. Fish Shellfish Immunol..

[128] Powell D.B., Ostrander G.K. (2000). Immune system. The Laboratory Fish.

[129] Ferguson H.W. (2006). Systemic Pathology of Fish.

[130] Haugarvoll E., Bjerkas I., Nowak B.F., Hordvik I., Koppang E.O. (2008). Identification and characterization of a novel intraepithelial lymphoid tissue in the gills of Atlantic salmon. J. Anat..

[131] Gomez D., Sunyer J.O., Slina I. (2013). The mucosal immune system of fish: The evolution of tolerating commensals while fighting pathogens. Fish Shellfish Immunol..

[132] Borgen H., Koppang E.O. (2021). Anatomy of teleost fish immune structures and organs. Immunogenetics.

[133] Castro R., Tafalla C., Beck P., Peatman E. (2015). Overview of fish immunity. Mucosal Health in Aquaculture.

[134] Barraza F., Montero R., Wong-Benito V., Valenzuela H., Godoy-Guzman C., Guzman F., Köllner B., Wang T., Secombes C.J., Maisey K. (2021). Revisiting the teleost thymus: Current knowledge and future perspectives. Biology.

[135] Rombout J.H., Yang G., Kiron V. (2014). Adaptive immune responses at mucosal surfaces of teleost fish. Fish Shellfish Immunol..

[136] Aas I.B., Austbo L., Falk K., Hordvik I., Koppang E.O. (2017). The interbranchial lymphoid tissue likely contributes to immune tolerance and defense in the gills of Atlantic salmon. Dev. Comp. Immunol..

[137] Möller A.M., Köllner B., Schmidt-Posthaus H., Segner H. (2014). The teleostean liver as an immunological organ: Intrahepatic immune cells (IHIC) in healthy and benzo(a)pyrene challenged rainbow trout (*Oncorhynchus mykiss*). Dev. Comp. Immunol..

[138] Ye R.R., Peterson D.R., Seemann F., Kitamura S.I., Lee J.S., Lau T.C.K., Tsui S.K.W., Au D.T.W. (2017). Immune competence assessment in marine medaka (*Oryzias melastigma*)—A holistic approach for immunotoxicology. Environ. Sci. Poll. Res..

[139] Zapata A., Chiba A., Vras A. (1996). Cells and tissues of the immune system of fish. The Fish Immune System: Organism, Pathogens, Environment.

[140] Yamauchi M., Kim E.Y., Iwata H., Tanabe S. (2005). Molecular characterization of the aryl hydrocarbon receptor (AHR1 and AHR2) from red seabream (*Pagrus major*). Comp. Biochem. Physiol. C.

[141] Hansson M.C., Wittzell H., Persson K., von Schantz T. (2004). Unprecedented genomic diversity of AhR1 and AhR2 genes in Atlantic salmon (*Salmo salar* L.). Aquat. Toxicol..

[142] Hansson M.C., Hahn M.E. (2008). Functional properties of the four Atlantic salmon (*Salmo salar*) aryl hydrocarbon receptor type 2 (AHR2) isoforms. Aquat. Toxicol..

[143] Lu M., Chang Z., Bae M.J., Oh S.M., Chung K.H., Park J.S. (2013). Molecular characterization of the aryl hydrocarbon receptor (AhR) pathway in goldfish (*Carassius auratus*) exposure to TCDD: The mRNA and protein levels. Fish Shellfish Immunol..

[144] Abnet C.C., Tanguay R.L., Hahn M.E., Heideman W., Peterson R.E. (1999). Two forms of aryl hydrocarbon receptor type 2 in rainbow trout (*Oncorhynchus mykiss*): Evidence for differential expression and enhancer specificity. J. Biol. Chem..

[145] Phalen L.J., Köllner B., Leclair L.A., Hogan N.S., van den Heuvel M.R. (2014). The effects of benzo(a)pyrene on leukocyte distribution and antibody response in rainbow trout (*Oncorhynchus mykiss*). Aquat. Toxicol..

[146] Song J.Y., Casanova-Nakayama A., Möller A.M., Kitamura S.I., Nakayama K., Segner H. (2020). Aryl hydrocarbon receptor signaling is functional in immune cells of rainbow trout (*Oncorhynchus mykiss*). Int. J. Mol. Sci..

[147] Whitehead A., Clark B.W., Reid N.M., Hahn M.E. (2016). When evolution is the solution to pollution: Key principles, and lessons from repeated adapation of killifish (*Fundulus heteroclitus*) populations. Evol. Appl..

[148] Powell W.H., Bright R., Bello S.M., Hahn M.E. (2000). Developmental and tissue-specific expression of AHR1, AHR2 and ARNT in dioxin-sensitive and–resistant populations of the marine fish *Fundulus heteroclitus*. Toxicol. Sci..

[149] Aranguren-Abadia L., Donald C.E., Eilertsen M., Gharbi N., Tronci V., Sorhus E., Mayer P., Nilsen T.O., Meier S., Goksoyr A. (2020). Expression and localization of the aryl hydrocarbon receptor and cytochrome P4501A during early development of Atlantic cod (*Gadus morhua*). Aquat. Toxicol..

[150] Holen E., Olsvik P.A. (2014). Aryl hydrocarbon receptor protein and CYP1A gene induction by LPS and phenanthrene in Atlantic cod (*Gadus morhua*) head kidney cells. Fish Shellfish Immunol..

[151] Nakayama A., Riesen I., Köllner B., Eppler E., Segner H. (2008). Surface marker-defined head kidney granulocytes and B-lymphocytes of rainbow trout express benzo[a]pyrene-inducible cytochrome P4501A protein. Toxicol. Sci..

[152] Phalen L.J., Köllner B., Hogan N.S., van den Heuvel M.R. (2017). Transcriptional responss in rainbow trout (*Oncorhynchus mykiss*) B cells and thrombocytes following in vivo exposure to benzo(a)pyrene. Environ. Toxicol. Pharmacol..

[153] Köllner B., Fischer U., Rombout J.H., Taverne-Thiele J.J., Hansen J.D. (2004). Potential involvement of rainbow trout thrombocytes in immune functions: A study using a panel of monoclonal antibodies and RT-PCR. Dev. Comp. Immunol..

[154] Nagasawa T., Nakayasu C., Rieger A.M., Barreda D.R., Somamoto T., Nakao M. (2014). Phagopcytosis by thrombocytes is a conserved innate immune mechanism in lower vertebrates. Front. Immunol..

[155] Holen E., Olsvik P.A. (2016). β-naphthoflavone interferes with cyp1c1, cox-2 and IL-8 gene transcription and leukotriene B4 secretion in Atlantic cod (*Gadus morhua*) head kidney cells during inflammation. Fish Shellfish Immunol..

[156] He R., Zhao L., Xu X., Zheng W., Zhang J., Zhang J. (2020). Aryl hydrocarbon receptor is required for immune response in *Epinephelus coioides* and *Danio rerio* infected with *Pseudomonas plecoglossidica*. Fish Shellfish Immunol..

[157] Husoy A.M., Myers M.S., Willi M.L., Collier T.K., Celander M., Goksoyr A. (1994). Immunohistochemical localization of CYP1A and CAP3A-like isozymes in hepatic and extrahepatic tissues of Atlantic cod (*Gadus morhua*), a marine fish. Toxicol. Appl. Pharmacol..

[158] Grinwis G.C., Besselink H.T., Van den Brandhof E.J., Bulder A.S., Engelsma M.Y., Kuiper R.V., Wester P.W., Vaal M.A., Vethaak A.D., Vos J.G. (2000). Toxicity of TCDD in European flounder *(Platichthys flesus*) with emphasis on histopathology and cytochrome P4501A induction in several organ systems. Aquat. Toxicol..

[159] Grinwis G.C., van den Brandhof E.J., Engelsma M.Y., Kuiper R.V., Vaal M.A., Vethaak A.D., Wester P.G., Vos J.G. (2001). Toxicity of PCB 126 in European flounder (*Platichthys flesus*) with emphasis on histopathology and cytochrome P4501A induction in several organ systems. Arch. Toxicol..

[160] Carlson E.A., Li Y., Zelikoff J.T. (2004). Benzo[a]pyrene-induced immunotoxicity in Japanese medaka (*Oryzias latipes*): Relationship between lymphoid CYP1A activity and humoral immune suppression. Toxicol. Appl. Pharmacol..

[161] Maisey K., Montero R., Corripio-Miyer Y., Toro-Ascuy D., Valenzuela B., Reyes-Cerpa S., Sandino A.M., Zou J., Wang T., Secombes C.J. (2016). Isolation and characterization of salmonid CD4+ T cells. J. Immunol..

[162] Tafalla C., Gonzalez L., Castro R., Granja A.G. (2017). B cell-activating factor regulates different aspects of B cell functionality and is produced by a subset of splenic B cells in teleost fish. Front. Immunol..

[163] Wang F., Hu C.B., Ma J.X., Gao K., Xiang L.X., Shao J.Z. (2017). Characterization of γδ T cells from zebrafish provides insights into their important role in adaptive humoral immunity. Front. Immunol..

[164] Niu J., Huang Y., Liu X., Zhang Z., Tang J., Wang B., Lu Y., Cai J., Jian J. (2020). Single cell RNA-seq reveals different subsets of non-specific cytotoxic cells in teleosts. Genomics.

[165] Yamaguchi T., Takizawa F., Furihata M., Soto-Lampe V., Dijkstra J.M., Fischer U. (2019). Teleost cytotoxic T cells. Fish Shellfish Immunol..

[166] Valdez Domingos F.X., Oliveira Ribeiro C.A., Pelletier E., Rouleau C. (2011). Tissue distribution and depuration kinetics of waterborne 14C-labeled light PAHs in mummichog (*Fundulus heteroclitus*). Environ. Sci. Technol..

[167] Möller A.M., Hermsen C., Floehr T., Lamoree M.H., Segner H. (2014). Tissue-specific metabolism of benzo(a)pyrene in rainbow trout (*Oncorhynchus mykiss*)—A comparison between liver and immune organs. Drug Metabol. Dispos..

[168] Logan D.T. (2007). Perspective on ecotoxicology of PAHs in fish. Hum. Ecol. Risk Assess..

[169] Quabius E.S., Krupp G., Secombes C.J. (2005). Polychlorinated biphenyl 126 affects expression of genes involved in stress-immune interaction in primary cultures of rainbow trout anterior kidney cells. Environ. Toxicol. Chem..

[170] Nakayama K., Kitamura S., Murakami Y., Song J., Jung S., Oh M., Iwata H., Tanabe S. (2008). Toxicogenomic analysis of immune-related genesin Japanese flounder (*Paralichthys olivaceus*) exposed to heavy oil. Mar. Poll. Bull..

[171] Hur D., Jeon J.K., Hong S. (2013). Analysis of immune gene expression modulated by benzo(a)pyrene in head kidney of olive flounder (*Paralichthys olivaceus*). Comp. Biochem. Physiol. B.

[172] Liu Q., Spitsbergen J.M., Cariou R., Huang C.Y., Jiang N., Goetz G., Hutz R.J., Tonellato P.J., Carvan M.J. (2014). Histopathologic alterations associated with global gene expression due to chronic dietary TCDD exposure in juvenile zebrafish. PLoS ONE.

[173] Egimezer G., Üstündag Ü.V., Ates P.S., Ünal F.D., Alturfan A.A., Emekli-Alturfan E., Altinoz M.A., Elmaci I. (2020). Methylnitrosurea, dimethylbenzanthracene and benzo(a)pyrene differentially affect refox pathways, apoptosis and immunity in zebrafish. Hum. Exp. Toxicol..

[174] Krasnov A., Koskinen H., Rexroad C., Afanasyev S., Mölsä H., Oikari A. (2005). Transcriptome responses to carbon tetrachloride and pyrene in the kidney and liver of juvenile rainbow trout (*Oncorhynchus mykiss*). Aquat. Toxicol..

[175] Mehinto A.C., Hampton L.M.T., Vidal-Dorsch D.E., Garcia-Reyero N., Arick M.A., Maruya K.A., Lao W., Vulpe C.D., Brown-Augustine M., Luguinov A. (2021). Transcriptomic respons patterns of hornyhead turbot (*Pleuronichthys verticalis*) dosed with polychlorinated biphenyls and polybrominated biphenyl ethers. Comp. Biochem. Physiol. D.

[176] Rogers M.L., Serafin J., Sepulveda M.S., Griffit R.J. (2021). The impact of salinity and dissolved oxygen regimes on transcriptomic immune responses to oil in early life stage *Fundulus grandis*. Comp. Biochem. Physiol. D.

[177] Curtis L.R., Bravo F., Bayne C.J., Tilton F., Arkoosh M.R., Lambertini E., Loge F.J., Collier T.K., Meador J.P., Tilton S.C. (2017). Transcriptional changes in innate immune genes in head kidneys from *Aeromonas salmonicida*-challenged rainbow trout fed a mixture of polycyclic aromatic hydrocarbons. Ecotoxicol. Environ. Saf..

[178] Li Z.H., Xu H., Zheng W., Lam S.H., Gong Z. (2013). RNA-sequencing analysis of TCDD-induced responses in zebrafish liver reveals high relatedness to in vivo mammalian models and conserved biological pathways. PLoS ONE.

[179] Liu Q., Rise M.L., Spitsbergen J.M., Hori T.S., Mieritz M., Geis S., McGraw J.E., Goetz G., Larson H., Hutz R.J. (2013). Gene expression and pathological alterations in juvenile rainbow trout due to chronic dietary TCDD exposure. Aquat. Toxicol..

[180] Volz D.C., Hinton D.E., Law J.M., Kullman S.W. (2006). Dynamic gene expression changes precede dioxin-induced liver pathogenesis in medaka fish. Toxicol. Sci..

[181] Williams T.D., Turan N., Diab A.M., Wu H., Mackenzie C., Bartie K.L., Hrydziuszko O., Lyons B.P., Stentiford G.D., Herbert J.M. (2011). Towards a system level understaning of non-model organisms samples from the environment: A network biology approach. PLoS Comput. Biol..

[182] Hogan N.S., Lee K.S., Köllner B., van den Heuvel M.R. (2010). The effects of the alkyl polycyclic aromatic hydrocarbon retene on rainbow trout (*Oncorhynchus mykiss*) immune response. Aquat. Toxicol..

[183] Song J.Y., Ohta S., Nakayama K., Murakami Y., Kitamura S.I. (2012). A time course study of immune response in Japanese flounder (*Paralichtyhs olivaceus*) exposed to heavy oil. Environ. Sci. Poll. Res..

[184] Li Z., Liang X., Liu W., Zhao Y., Yang H., Li W., Adamovsky O., Martyniuk C.J. (2020). Elucidating mechanisms of immunotoxicity by benzotriazole ultraviolet stabilizers in zebrafish (*Danio rerio*): Implication of the AHR-IL17/IL-22 immune pathway. Environ. Poll..

[185] Tian J., Rabson A.B., Gallo M.A. (2002). Ah receptor and NF-κB interactions: Mechanisms and physiological implications. Chem. Biol. Interact..

[186] Arkoosh M.R., Clemons E., Huffman P., Sanborn H.R., Casillas E., Stein J.E. (1996). Leukoproliferative response of splenocytes from English sole (*Pleuronectes vetulus*) exposed to chemical contaminants. Environ. Toxicol. Chem..

[187] Hart L.J., Smith S.A., Smith B.J., Robertson J. (1998). Subacute immunotoxic effects of the polycyclic aromatic hydrocarbon 7,12-dimethylbenzanthracene (DMNA) on spleen and pronephros leukocytioc cell counts and phagocytic cell activity in tilapia (*Oreochromis niloticus*). Aquat. Toxicol..

[188] Carlson E.A., Li Y., Zelikoff J.T. (2002). Exposure of Japanese medaka (*Oryzias latipes*) to benzo(a)pyrene suppresses immune function and host resistance against bacterial challenge. Aquat. Toxicol..

[189] Duffy J.E., Zelikoff J.T. (2006). The relationship between noncoplanar PCB-induced immunotoxicity and hepatic CYP1A induction in a fish model. J. Immunotoxicol..

[190] Xu H., Zhang X., Li H., Li C., Huo X.J., Hou L.P., Gong Z. (2018). Immune response induced by major environmental pollutants through altering neutrophils in zebrafish larvae. Aquat. Toxicol..

[191] Qamar A., Waheed J., Zhang Q.H., Namula Z., Chen Z., Chen J.J. (2020). Immunotoxicological effects of dioxin-like polychlorinated biphenyls extracted from Zhanjiang Bay sediments in zebrafish. Environ. Monit. Assess..

[192] Karrow N.A., Bols N.C., Whyte J.J., Solomon K.R., Dixon D.G., Boermans H.J. (2001). Effects of creosote exposure on rainbow trout pronephros phagocytic activity and the percentage of lymphoid B cells. J. Toxicol. Environ. Health A.

[193] Danion M., LeFloch S., Kanan R., Lamopur F., Quentel C. (2011). Effects of in vivo chronic hydrocarbons on sanitary status and immune system of sea bass (*Dicentrarchus labrax*). Aquat. Toxicol..

[194] Leclair L.A., MacDonald G.T., Phalen L.J., Köllner B., Hogan N.S., van den Heuvel M.R. (2013). The immunological effects of water-borne oil sands-surface waters derived napthenic acids on rainbow trout (*Oncorhynchus mykiss*). Aquat. Toxicol..

[195] Omar-Ali A., Hohn C., Allen P.J., Rodriguez J., Petrie-Hanson L. (2015). Tissue PAH, blood cell and tissue changes following exposure to water accommodated fractions of crude oil in Alligator gar, *Atratosteus spatula*. Mar. Environ. Res..

[196] Iwanowicz L.R., Lerner T.D., Blazer V.S., McCormick S.D. (2005). Aqueous exposure to Aroclor 1254 modulates the mitogenic response of Atlantic salmon anterior kidney T cells: Indications of short- and long-term immunomodulation. Aquat. Toxicol..

[197] Reynaud S., Deschaud P. (2005). The effects of 3-methylcholanthrene on lymphocyte proliferation in the common carp (*Cyprinus carpio*). Toxicology.

[198] Holladay S.D., Smith S.A., Besteman E.G., Deyab A.S., Gogal R.M., Hrubec T., Robertson J.L., Ahmed S.A. (1998). Benzo(a)pyrene-induced hypocellularity of the pronephros in tilapia (*Oreochromis niloticus*) is accompanied by alterations in stromal and parenchymal cells and by enhanced immune cell apoptosis. Vet. Immunol. Immunopathol..

[199] Reynaud S., Duchiron C., Deschaux P. (2004). 3-methylcholanthrene induces lymphocyte and phagocyte apoptosis in common carp (*Cyprinus carpio*) in vitro. Aquat. Toxicol..

[200] Sweet L.I., Passino-Reader D.R., Meier P.G., Omman G.M. (1998). Fish thymocyte viability, apoptosis, and necrosis: In vitro effects of organochlorine contaminants. Fish Shellfish Immunol..

[201] Spitsbergen J.M., Schat K.A., Kleeman J.M., Peterson R.E. (1986). Interactions of 2,3,7,8-tetrachlorodibenzo-p-dioxin with immune responses of rainbow trout. Vet. Immunol. Immunopathol..

[202] Rice C.D., Schlenk D. (1995). Immune function and cytochrome P4501 activity after acute exposure to 3,3′,4,4′,5-pentachlorobiphenyl (PCB 126) in channel catfish. J. Aquat. Anim. Health.

[203] Hutchinson T.H., Field M.D.R., Manning M.J. (1999). Evaluation of immune function in juvenile turbot *Scophthalmus maximus* (L.) exposed to sediments contaminated with polychlorinated biphenyls. Fish Shellfish Immunol..

[204] Palm R.C., Powell D.B., Skillman A., Godtfredsen K. (2003). Immunocompetence of juvenile chinook salmon against *Listonella anguillarum* following dietary exposure to polycyclic aromatic hydrocarbons. Environ. Toxicol. Chem..

[205] Iwanowicz L.R., Blazer V.S., McCormick S.D., vanVeld P.A., Ottinger C.A. (2009). Arocolor 1248 exposure leads to immunomodulation, decreased disease resistance and endocrine disruption in the brown bullhead, *Ameiurus nebulosus*. Aquat. Toxicol..

[206] Song J.Y., Nakayama K., Murakami Y., Jung S.J., Oh M.J., Matsuoka S., Kawakami H., Kitamura S.I. (2008). Does heavy oil pollution induce bacterial diseases in Japanese flounder *Paralichthys olivaceus*?. Mar. Poll. Bull..

[207] Swanyna J.M., Spivia W.R., Radecki K., Fraser D.A., Lowe C.G. (2017). Association between chronic organochlorine exposure and immunotoxicity in the round stingray (*Urobatis halleri*). Environ. Poll..

[208] Steinel N.C., Bolnick D.I. (2017). Melanomacrophage centers as a histological indicator of immune function in fish and other poikilotherms. Front. Immunol..

[209] Payne J.F., Fancey L.F. (1989). Effect of polycyclic aromatic hydrocarbons on immune responses in fish: Change in melanomacrophage centers in flounder (*Pseudopleuronectes americanus*) exposed to hydrocarbon-contaminated sediments. Mar. Environ. Res..

[210] Van der Weiden M.E.J., van der Kolk J., Seinen W., van den Berg M. (1992). Concurrence of P450 1A1 induction and toxic effects after administration of a low dose of 2,3,7,8-tetrachlordibenzo-p-dioxin (TCDD) in rainbow trout (*Oncorhynchus mykiss*). Aquat. Toxicol..

[211] Dalmo R.A., Ingebrigsten K., Bogwald J. (1997). Non-specific defense mechanisms in fish, with particular reference to the reticulo-endothelial system (RES). J. Fish Dis..

[212] Bols N.C., Brubacher J.L., Ganassin R.C., Lee L.E.J. (2001). Ecotoxicology and innate immunity in fish. Dev. Comp. Immunol..

[213] Smolowitz R.M., Hahn M.E., Stegeman J.J. (1991). Immunocytochemical localization of cytochrome P4501A1 induced by 3,3‘,4,4‘-tetrachlorobiphenyl and 2,3,7,8-tetrachlorodibenzofuran in liver and extrahepatic tissues of the teleost *Stenostomus chrysops* (scup). Drug Metab. Dispos..

[214] Sarasquete C., Segner H. (2000). Cytochrome P4501A (CYP1A) in teleostean fishes. A review of immunohistochemical studies. Sci. Total Environ..

[215] Lemaire-Gony S., Lemaire P., Pulsford A.L. (1995). Effects of cadmium and benzo(y)pyrene on the immune system, gill ATPase and EROD activity of European seabass *Dicentrarchus labrax*. Aquat. Toxicol..

[216] Seeley K.R., Weeks-Perkins B.A. (1997). Suppression of natural cytotoxic cells and macrophage phagocytic function in oyster toadfish exposed to 7,12-dimethylbenz(a)anthracene. Fish Shellfish Immunol..

[217] Regala R.P., Rice C.D., Schwedtke C.E., Dorciak I.R. (2001). The effects of tributyltin (TBT) and 3,3′,4,4′,5-pentachlorbiphenyl (PCB-126) on antibody response and phagocyte oxidative burst activity in channel catfish. Arch. Environ. Contam. Toxicol..

[218] Hutchinson T.H., Field M., Manning M.J. (2003). Evaluation of non-specific immune functions in dab, *Limanda limanda* L., following short-term exposure to sediments contaminated with polyaromatic hydrocarbons and/or polychlorinated biphenyls. Mar. Environ Res..

[219] Kennedy C.J., Farell A.P. (2008). Immunological alterations in juvenile Pacific herring, *Clupea pallasi*, exposed to aqueous hydrocarbons derived from crude oil. Environ. Poll..

[220] Arkoosh M.R., Clemons E., Myers M.S., Casillas E. (1994). Suppression of B cell-mediated immunity in juvenile Chinook salmon (*Oncorhynchus tshawytscha*) after exposure to either a polycyclic aromatic hydrocarbon or to polychlorinated biphenyls. Immunopharmacol. Immunotoxicol..

[221] Jacobson K.C., Arkoosh M.R., Kagley A.N., Clemons E.R., Collier T.K., Casillas E. (2003). Cumulative effects of natural and anthropogenic stress on immune function and disease resistance in juvenile Chinook salmon. J. Aquat. Anim. Health.

[222] White S.L., DeMario D.A., Iwanowicz L.R., Blazer V.S., Wagner T. (2020). Tissue distribution and immunomodulation in channel catfish (*Ictalurus punctatus*) following dietary exposure to polychlorinated biphenyl aroclors and food deprivation. Int. J. Environ. Res. Public health.

[223] Smith D.A., Schurig G.G., Smith S.A. (1999). The hemolytic plaque-forming cell assay in tilapia (*Oreochromis niloticus*) exposed to benzo[a]pyrene: Enhanced or depressed plaque formation depends on dosing schedule. Toxicol. Methods.

[224] Tahir A., Fletcher T.C., Houlihan D.F., Secombes C.J. (1993). Effect of short-term, exposure to oil-contaminated sediments on the immune response of dab, *Limanda limanda* (L.). Aquat. Toxicol..

[225] Bado-Nilles A., Quentel C., Mazurais D., Zambonino-Infante J.L., Auffret M., Thomas-Guyon H., Le Floch S. (2011). In vivo effects of the soluble fraction of light cycle oil on immune functions in the European sea bass, *Dicentrachus labrax* (Linné). Ecotoxicol. Environ. Saf..

[226] Winkelhake J.L., Vodicnik M.J., Taylor J.L. (1983). Induction in rainbow trout of an acute phase (C-reactive) protein by chemicals of environmental concern. Comp. Biochem. Physiol. C.

[227] Duffy J.E., Carlson E., Li Y., Zelikoff J.T. (2003). Age-related differences in the sensitivity of the immune response to a coplanar PCB. Ecotoxicology.

[228] Martin S.A.M., Douglas A., Houlihan D.F., Secombes C.J. (2010). Starvation alters the liver transcriptome of the innate immune response of Atlantic salmon (*Salmo salar*). BMC Genom..

[229] Köllner B., Wasserrab B., Kotterba G., Fischer U. (2002). Evaluation of immune functions of rainbow trout (*Oncorhynchus mykiss*)—how can environmental influences be detected?. Toxicol. Lett..

[230] Segner H., Wenger M., Möller A.M., Köllner B., Casanova-Nakayama A. (2012). Immunotoxic effects of environmental toxicants in fish—How to assess them?. Environ. Sci. Poll. Res..

[231] Nacci D., Huber M., Champlin D., Jayaraman S., Cohen S., Gauger E., Fong A., Gomez-Chiarri M. (2009). Evolution of tolerance to PCBs and susceptibility to a bacterial pathogen (*Vibrio harveyi*) in Atlantic killifish (*Fundulus heteroclitus*) from New Bedford (MA, USA) harbor. Environ. Pollut..

[232] Ruggeri P., Du X., Crawford D.L., Oleksiak M.F. (2019). Evolutionary toxicogenomics of the striped killifish (*Fundulus majalis*) in the New Bedford Harbor (Massachusetts, USA). Int. J. Mol. Sci..

[233] Spitsbergen J.M., Kleeman J.M., Peterson R.E. (1988). 2,3,7,8-tetrachlorodibenzo-p-dioxin toxicity in yellow perch (*Perca flavescens*). J. Toxicol. Environ. Health.

[234] Spitsbergen J.M., Kleeman J.M., Peterson R.E. (1988). Morphological lesions and acute toxicity in rainbow trout (*Salmo gairdneri*) treated with 2,3,7,8-tetrachlorodibenzo-p-dioxin. J. Toxicol. Environ. Health.

[235] Walter G.L., Jones P.D., Giesy J.P. (2000). Pathologic alterations in adult rainbow trout, *Oncorhynchus mykiss*, exposed to dietary 2,3,7,8-tetrachlorodibenzo-p-dioxin. Aquat. Toxicol..

[236] Gao Y., Xu H., Li L., Niu C. (2020). Immune defense parameters of wild fish as sensitive biomarkers for ecological risk assessment in shallow sea ecosystems: A case study with wild mullet (*Liza haematocheila*) in Liaodong Bay. Ecotoxicol. Environ. Saf..

[237] Bravo C.F., Curtis L.R., Myers M.S., Meador J.P., Johnson L.L., Buzitis J., Collier T.K., Morrow J.D., Laetz C.A., Loge F.J. (2011). Biomarker responses and disease susceptibility in juvenile rainbow trout *Oncorhynchus mykiss* fed a high molecular weight PAH mixture. Environ. Toxicol. Chem..

[238] Maule A.G., Jorgensen E.H., Vijayan M.M., Killie J.A.E. (2005). Aroclor 1254 exposure reduces disease resistance and innate immune responses in fasted Arctic Charr. Environ. Toxicol. Chem..

[239] Ekman E., Akerman G., Balk L., Norrgren L. (2004). Impact of PCB on resistance to *Flavobacterium psychophrilum* after experimental infection of rainbow trout *Oncorhynchus mykiss* eggs by nanoinjection. Dis. Aquat. Org..

[240] Song J.Y., Nakayama K., Murakami Y., Kitamura S.I. (2011). Heavy oil exposure induces high mortalities in virus carrier Japanese flounder *Paralichthys olivaceus*. Mar. Poll. Bull..

[241] Lundin J.I., Spromberg J.A., Jorgensen J.C., Myers M.S., Chittaro P.M., Zabel R.W., Johnson L.L., Neely R.M., Scholz N.L. (2019). Legacy habitat contamination as a limiting factor for Chinook salmon recovery in the Willamette Basin, Oregon, USA. PLoS ONE.

[242] Barron M.G. (2012). Ecological impacts of the Deepwater Horizon oil spill: Implications for immunotoxicity. Toxicol. Pathol..

[243] Murawski S.A., Hogarth W.T., Peebles E.B., Barberi L. (2014). Prevalence of external skin lesions and polycyclic aromatic hydrocarbon concentrations in Gulf of Mexico fishes, post-Deepwater Horizon. Trans. Am. Fish. Soc..

[244] Bayha K.M., Ortell N., Ryan C.N., Griffit K.J., Krasnec M., Sena J., Ramaraj T., Takeshita R., Mayer G.D., Schilkey F. (2017). Crude oil impairs immune function and increases susceptibility to pathogenic bacteria in southern flounder. PLoS ONE.

[245] Jones E.R., Martyniuk C.J., Morris J.M., Krasnec M.O., Griffitt R.J. (2017). Exposure to Deepwater Horizon oil and Corexit 9500 at low concentrations induces transcriptional changes and alters immune transcriptional pathways in sheephead minnows. Comp. Biochem. Physiol. D.

[246] Rodgers M.L., Takeshita R., Griffitt R.J. (2018). Deepwater Horizon oil alone and in conjunction with *Vibrio anguillarum* exposure modulates immune response and growth in red snapper (*Lutjanus campechanus*). Aquat. Toxicol..

[247] Schlezinger J.J., Blickarz C.E., Mann K.K., Doerre S., Stegeman J.J. (2000). Identification of NF-κB in the marine fish *Stenostomus chyrsops* and examination of its activation by aryl hydrocarbon receptor agonists. Chem. Biol. Interact..

[248] Reynaud S., Deschaux P. (2006). The effects of polycyclic aromatic hydrocarbons on the immune system in fish: A review. Aquat. Toxicol..

[249] Reynaud S., Duchiron C., Deschaux P. (2003). 3-methylcholanthrene inhibits lymphocyte proliferation and increases in intracellular calcium levels in common carp (*Cyprinus carpio* L.). Aquat. Toxicol..

[250] Hahn M.E. (2011). Mechanistic research in aquatic toxicology: Perspectives and future directions. Aquat. Toxicol..

[251] Segner H. (2011). Moving beyond a descriptive aquatic toxicology: The value of biological process and trait information. Aquat. Toxicol..

[252] Ankley G.T., Benett R.S., Erickson R.J., Hoff D.J., Hornung M.W., Johnson R.D., Mount D.R., Nichols J.W., Russom C.L., Schmieder P.K. (2011). Adverse outcome pathways: A conceptual framework to support ecotoxicology research and risk assessment. Environ. Toxicol. Chem..

[253] Brown-Peterson N.J., Krasnec M., Takeshita R., Ryan C.N., Griffit K.J., Lay C., Mayer G.D., Bayha K.M., Hawkins W.E., Lipton I. (2015). A multiple endpoint analysis of the effects of chronic exposure to sediment contaminated with Deepwater Horizon oil on juvenile Southern flounder and their associated microbiomes. Aquat. Toxicol..

[254] Sun Y., Tang L., Liu Y., Zhou B.S., Lam P.K.S., Lam J.W.C., Chen L. (2019). Activation of aryl hydrocarbon receptor by dioxin directly shifts gut microbiota in zebrafish. Environ. Poll..

[255] DeBofsky A., Xie Y., Grimard C., Alcaraz A.J., Brinkmann M., Hecker M., Giesy J.P. (2020). Differential responses of gut microbiota of male and female fathead minnow (*Pimephales promelas*) to a short-term environmentally relevant, aqueous exposure to benzo(a)pyrene. Chemosphere.

[256] Hu C., Liu M., Wan T., Tang L., Sun B., Zhou B., Lam J.W.C., Lam P.K.S., Chen L. (2021). Disturbances in microbial and metabolic communication across the gut-liver axis induced by a dioxin-like pollutant: An integrated metagenomics and metabolomics analysis. Environ. Sci. Technol..

[257] Redfern L.K., Jayasundra N., Singleton D.R., Di Giulio R.T., Carlson J., Summer S.J., Gunsch C.K. (2021). The role of the gut microbial community and metabolomic shifts in adaptive resistance of Atlantic killifish (*Fundulus heteroclitus*) to polycyclic aromatic hydrocarbons. Sci. Total Environ..

[258] Kau A.L., Ahern P.P., Griffin N.W., Goodman A.L., Gordon J.I. (2011). Human nutrition, the gut microbiome and the immune system. Nature.

[259] McDermott A.J., Huffnagle G.B. (2014). The microbiome and regulation of mucosal immunity. Immunology.

[260] Tuddenham S., Sears C.L. (2015). The intestinal microbiome and health. Curr. Opin. Infect. Dis..

[261] Kataoka C., Kashiwada S. (2021). Ecological risks due to immunotoxicological effects on aquatic organisms. Int. J. Mol. Sci..

[262] Sniezko S.F. (1974). The effects of environmental stress on outbreaks of infectious diseases of fishes. J. Fish Biol..

[263] Thrush M.A., Murray A.G., Brun E., Wallace S., Peeler E.J. (2011). The application of risk and disease modelling to emerging freshwater diseases in wild aquatic animals. Freshw. Biol..

[264] Rohr J.R., Palmer B.D. (2013). Climate change, multiple stressors, and the decline of ectotherms. Conserv. Biol..

[265] Whitehead A. (2013). Interactions between oil-spill pollutants and natural stressors can compound ecotoxicological effects. Integr. Comp. Biol..

[266] Segner H., Schmitt-Jansen M., Sabater S. (2014). Assessing the impact of multiple stressors on aquatic biota: The receptor’s side matters. Environ. Sci. Technol..

[267] Fey S.B., Siepielski A.M., Nussle S., Cervantes-Yoshida K., Hwan J.L., Huber E.R., Fey M.J., Catenazzi A., Carlson S.M. (2015). Recent shifts in the occurrence, cause and magnitude of animal mass mortality events. Proc. Natl. Acad. Sci. USA.

[268] Groner M.L., Hoenig J.M., Pradel R., Choquet R., Vogelbein W.K., Gauthier T., Friedrichs M.A.M. (2018). Dermal mycobacteriosis and warming sea surface temperatures are associated with elevated mortality of striped bass in Chesapeake bay. Ecol. Evol..

